# Comparative anatomy and genetic bases of fruit development in selected Rubiaceae (Gentianales)

**DOI:** 10.1002/ajb2.1785

**Published:** 2021-10-26

**Authors:** Héctor Salazar‐Duque, Juan F. Alzate, Aura Urrea Trujillo, Cristina Ferrándiz, Natalia Pabón‐Mora

**Affiliations:** ^1^ Instituto de Biología Universidad de Antioquia Medellín Colombia; ^2^ Centro Nacional de Secuenciación Genómica–CNSG, Sede de Investigación Universitaria‐SIU Universidad de Antioquia Medellín Colombia; ^3^ Facultad de Medicina Universidad de Antioquia Medellín Colombia; ^4^ Instituto de Biología Molecular y Celular de Plantas Consejo Superior de Investigaciones Científicas‐Universidad Politécnica de Valencia Valencia Spain

**Keywords:** bHLH genes, epicarp, fruit diversity, MADS‐box genes, pericarp, *REPLUMLESS*, Rubiaceae

## Abstract

**Premise:**

The Rubiaceae are ideal for studying the diversity of fruits that develop from flowers with inferior ovary. We aimed to identify morpho‐anatomical changes during fruit development that distinguish those derived from the carpel versus the extra‐carpellary tissues. In addition, we present the fruit genetic core regulatory network in selected Rubiaceae species and compare it in terms of copy number and expression patterns to model core eudicots in the Brassicaceae and the Solanaceae.

**Methods:**

We used light microscopy to follow morphoanatomical changes in four selected species with different fruit types. We generated reference transcriptomes for seven selected Rubiaceae species and isolated homologs of major transcription factors involved in fruit development histogenesis, assessed their homology, identified conserved and new protein motifs, and evaluated their expression in three species with different fruit types.

**Results:**

Our studies revealed ovary‐derived pericarp tissues versus floral‐cup‐derived epicarp tissues. Gene evolution analyses of *FRUITFULL, SHATTERPROOF, ALCATRAZ, INDEHISCENT* and *REPLUMLESS* homologs suggest that the gene complement in Rubiaceae is simpler compared to that in Brassicaceae or Solanaceae. Expression patterns of targeted genes vary in response to the fruit type and the developmental stage evaluated.

**Conclusions:**

Morphologically similar fruits can have different anatomies as a result of convergent tissues developed from the epicarps covering the anatomical changes from the pericarps. Expression analyses suggest that the fruit patterning regulatory network established in model core eudicots cannot be extrapolated to asterids with inferior ovaries.

The Rubiaceae (Gentianales; asterids) is the fourth largest angiosperm family, occurring on all continents, with ca. 13,000 species circumscribed in three subfamilies, more than 40 tribes, and 620 genera (Wikström et al., 2015). The family presents outstanding variation in terms of carpel number, degree of fusion, and fruit types largely unexplored both morphologically and genetically. In the family, simple fruits that are dry dehiscent, drupaceous, or fleshy can occur (Bremer et al., 1996). The independent occurrence of dense‐headed inflorescences has also facilitated the development of multiple fruits, formed by the fusion of different flowers, like in the case of *Morinda citrifolia* (Morindeae) in the Rubioideae (Razafimandimbison et al., 2012). According to the most recent phylogenetic analyses, different fruit types have evolved independently several times with no less than 12 independent acquisitions of fleshy fruits within the family (Bremer and Eriksson, 1992; Bremer, 1996). In addition, analyses within specific clades suggest that fleshy fruits have evolved from dry fruits independently several times, as the earliest divergent groups in each subfamily are characterized by having capsules (Rova et al., 2002; Motley et al., 2005). Despite such diversity of fruit types in the family, few comparative studies have explored the morphoanatomical changes of the carpel to fruit transformation (Roth and Lindorf, 1971; Bremer and Eriksson, 2009; De Toni and Mariath, 2011; Giacomin, 2015), and not one study is available on the genes shaping such diversity.

One aspect that complicates the study of fruit diversity in the family is the generalized presence of inferior ovaries, as in most other Asterales (Endress, 2011; see exception on “secondarily superior” by Igersheim et al., 1994). In turn, the floral cup develops fused to the ovary wall. The common occurrence of inferior ovaries raises two difficulties, namely, the interpretation on the homology of the floral cup and the addition of extra‐carpellary tissue to late developmental processes like fruit development. The axial (axis‐derived) versus the appendicular (floral‐organ‐derived) nature of the cup, has been historically debated (Douglas, 1944, 1957 and references within), but in general the number, course, and splitting of the vascular rings, is used to determine whether the inferior ovary is surrounded by the axis or by the fused floral organs (Douglas et al., 1944, 1957; Puri et al., 1951; Fukuoka, 1980). However, this is not a straightforward task and has proven to be a complex feature to evaluate ontogenetically (Puri et al., 1951).

The genetic underpinnings of the carpel to fruit transformation are known from the model core eudicot *Arabidopsis thaliana* (Brassicaceae, Rosids). Fruits of *A. thaliana* are capsules with longitudinal dehiscence and a persistent medial replum, called siliques. The histogenesis and the resulting shattering in the *A. thaliana* fruit have been associated with the role of specific transcription factors. A first tier in the genetic regulation of fruit development is based on the tissue‐specific activation of *FRUITFULL* (*FUL*), *SHATTERPROOF 1* and *2* (*SHP 1*/*2*) and *REPLUMLESS* (*RPL*). *FUL* is responsible for proper cell division and proliferation in the valves, while *SHP 1* and *2* control the differentiation of the dehiscence zone (Gu et al., 1998; Ferrándiz et al., 1999; Liljegren et al., 2000, 2004). Both are members of the MADS‐box gene family (Ferrándiz et al., 1999; Liljegren et al., 2000, 2004). *RPL* controls the identity of the outer medial persistent tissue called the replum, and it belongs to the *BELL‐like* homeodomain gene lineage (Roeder et al., 2003). *FUL* negatively regulates *SHP* (Ferrándiz et al., 2000; Liljegren et al., 2000), and negative regulation of *RPL* and *FUL* together defines the expression layers of *SHP* (Roeder et al., 2003). *SHP* is a major regulator in the second tier of the genetic control in fruit histogenesis, as it interacts with *ALCATRAZ* (*ALC*) and *INDEHISCENT* (*IND*) to form the dehiscence zone (Alvarez and Smyth, 1999; Rajani and Sundaresan, 2001; Liljegren et al., 2004). While *IND* controls both the lignified and the separation layers, *ALC* maintains the parenchymatous nature of the separation layer (Alvarez and Smyth, 1999; Rajani and Sundaresan, 2001; Liljegren et al., 2004). Comparative analyses across Brassicaceae have suggested that shifts in the expression of the same transcription factors could control changes from dehiscent to indehiscent fruits in closely related species (Mühlhausen et al., 2013; Carey et al., 2019). However, the Brassicaceae have only dry fruits; hence, tissue specialization associated with fleshy fruit formation cannot be studied in this family.

Despite numerous plant families having diverse fruit types that can be comparatively studied from morphoanatomical and genetic perspectives, few have emerged successfully as systems where functional analyses have been implemented. For instance, the Solanaceae (Solanales, asterids) have been used as a reference family to study the genetic mechanisms underlying fruit type diversity because both dry and fleshy fruits are present among the members (Chung et al., 2010; Dong et al., 2013). Moreover, phylogenetic optimization of fruit types results in a single major transition from dry to fleshy fruits coinciding with the radiation of the Solanoideae (Knapp, 2002). Tobacco (*Nicotiana tabacum*) and tomato (*Solanum lycopersicum*) have served as model species for studying the function of the reference transcription factors in different fruit types of species sharing a recent common ancestor, with particular reference to ripening in tomato and dehiscence in tobacco (Seymour et al., 2013; Gómez et al., 2014; Garceau et al., 2017; Ballester and Ferrándiz, 2017). However, a whole‐genome multiplication (likely triplication) event resulted in numerous copies of each gene lineage and an added difficulty for expression and functional analysis of the fruit development genetic network (Tomato Genome Consortium, 2012; Kim et al., 2014; Ortíz‐Ramírez et al., 2018).

The Rubiaceae provides a unique framework for comparative developmental studies. The diversity of fruits in the family provides an appropriate system to implement developmental and evolutionary studies aimed to assess the underlying morphological and genetic mechanisms responsible for divergent fruit types in closely related species. Fruit development and ripening studies in the Rubiaceae are key to better understand the reproduction of important crops like *Coffea arabica* (coffee), *Borojoa patinoi* (borojo), and *Morinda citrifolia* (noni), with very different fruit types that have not been studied in a comparative evolutionary and developmental context. In this study, we specifically aimed to (1) document the morphoanatomical changes occurring during the carpel‐to‐fruit transformation in four species with different fruit types, (2) assess anatomical homologies and homoplasies, taking into account the spatial distribution of the carpellary versus the extracarpellary tissues, (3) record evidence in favor of either the axial (axis derived) versus appendicular (floral organ derived) nature of the floral cup, (4) identify gene homologs from major fruit patterning genes (i.e., *ALC, IND, FUL, RPL*, and *SHP*) in representative Rubiaceae species, and (5) compare the expression patterns of targeted genes in the three fruit types in Rubiaceae to better understand the functional fruit gene regulatory network in this plant family. We hope that this work will serve as a framework for morphoanatomical features of different fruit types in the family and for the basis of homolog identification of most transcription factors involved in fruit development in this specious family of flowering plants.

Here, we sampled selected species across the phylogeny, based on their fruit type. Specifically, we selected a member of the Cinchonoideae, *Cephalanthus occidentalis*, with thickened flowering heads and dry fruits; three members of the Ixoroideae, including *Condaminea corymbosa* with capsules, *Borojoa patinoi* with giant berries, and *Coffea arabica* with drupes; and three members of the Rubioideae including *Galium hypocarpium* with berries, *Palicourea angustifolia* with drupes, and *Morinda citrifolia* with multiple fleshy fruits (Figure 1). Our work provides (1) a comprehensive description of the morphoanatomical changes underlying the development of different fruit types in the family in four selected species (*C. corymbosa, G. hypocarpium, M. citrifolia*, and *P. angustifolia*), (2) the identification of gene homologs for major gene families involved in fruit histogenesis in the seven selected species of Rubiaceae for which de novo transcriptomes were generated, and (3) the assessment of target gene expression in three different fruit types.

**Figure 1:**
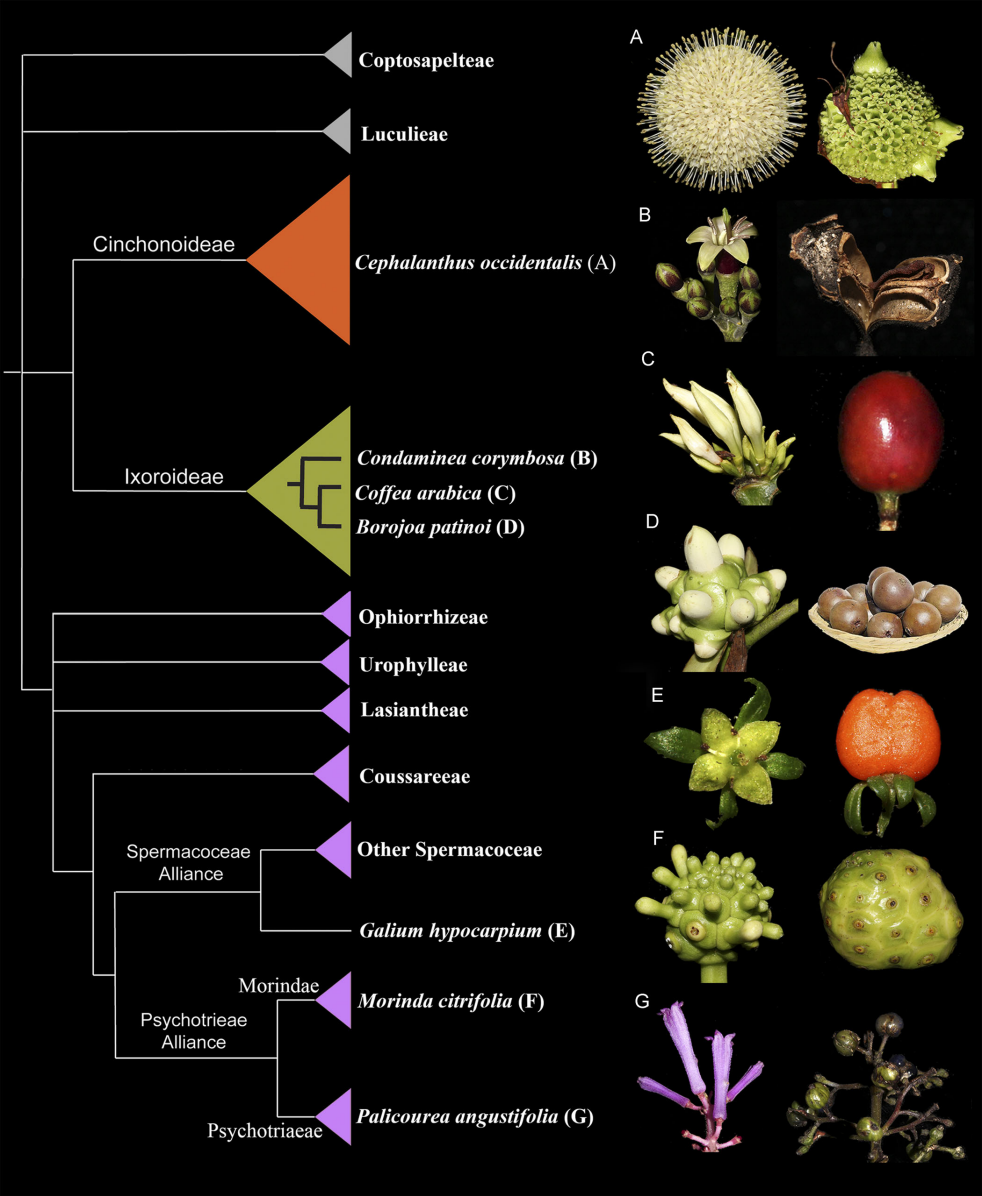
**(**Left) Summary tree of the phylogenetic relationships in Rubiaceae modified from Bremer and Eriksson (2009), highlighting the three major subfamilies: Cinchonoideae (orange), Ixoroideae (green), and Rubiaoideae (purple). (Right) Inflorescences, flowers, and fruits of the sampled species in this manuscript, labelled in the tree

## MATERIALS AND METHODS

### RNA extractions and the generation of reference transcriptomes

Reference transcriptomes from each species were generated from mixed material including leaves, floral buds, and young fruits. Species sampled included *Borojoa patinoi* Cuatrec. (Pabón‐Mora N & Salazar‐Duque H, *NP392*, Cocorná, Antioquia, Colombia), *Cephalanthus occidentalis L*. (Pabón‐Mora N, *NP445*, cultivated, Botanischer Garten, Dresden, Germany), *Coffea arabica* Benth. (Pabón‐Mora N & Salazar‐Duque H, *NP393*, Cocorná, Antioquia, Colombia), *Condaminea corymbosa* Ruiz & Pav. (Pabón‐Mora N & Salazar‐Duque H, *NP413*, Medellín, Antioquia, Colombia), *Galium hypocarpium* (L.) Forsberg. (Pabón‐Mora N & Salazar‐Duque H, *NP390*, Medellín, Antioquia, Colombia), *Morinda citrifolia* L. (Pabón‐Mora N & Salazar‐Duque H, *NP401*, Medellín, Antioquia, Colombia), and *Palicourea angustifolia* Kunth. (Pabón‐Mora N & Salazar‐Duque H, *NP414*, Santa Elena, Antioquia, Colombia). All vouchers are deposited in HUA (Medellín, Colombia).

Plant material derived from different flower to fruit developmental stages and from young leaves from one individual per species was ground in liquid nitrogen. Different methodologies for total RNA extraction were evaluated and standardized for each species. Different protocols including phenol–chloroform extraction, the use of Trizol reagent (Invitrogen, Carlsbad, CA, USA), CTAB with LiCl_2_ precipitation (Chang et al., 1993) and PureLink Plant RNA Reagent (Thermo Fisher Scientific, Carlsbad, CA, USA) were tested. The different protocols were used to minimize the impact of mucilage‐rich tissue and abundant secondary metabolites during RNA extraction. In general, only young leaves, floral buds, and fruits were included in the extractions, avoiding bracts, pedicels, anthetic flowers, and old fruits. The best‐performing protocol (i.e., the one that resulted in large amounts of RNA and low DNA or protein contamination) was PureLink Plant RNA Reagent. RNA quality was verified by spectrophotometry, by fluorometry, and by visual inspection of the ribosomal subunits separated electrophoretically in a 1.5% agarose gel.

The RNA‐seq libraries were done using a Truseq mRNA library construction kit (Illumina, San Diego, CA, USA) per species and sequenced in a HiSeq. 2000 Illumina instrument reading 100‐bp paired‐end reads. The transcriptomes were assembled de novo. Read cleaning was performed with PRINSEQ‐LITE with a quality threshold of Q35, and contig assembly was computed using the Trinity package v2.5.1 (https://github.com/trinityrnaseq/trinityrnaseq/wiki) and the default settings (Trinity –seqType fq –max_memory 250 G –CPU 40 –trimmomatic –full_cleanup). Transcriptome metrics can be found in Appendix S1.

### Fruit gene homologs isolation and phylogenetic analyses

BLASTN searches for each of the candidate genes were performed using the *Arabidopsis thaliana* canonical sequences *AGAMOUS*/*SHATTERPROOF, APETALA1*/*FRUITFULL, REPLUMLESS, ALCATRAZ*/*SPATULA* and *HECATE1*/*HEC2*/*HEC3*/*INDEHISCENT* as queries to identify homologues in the Rubiaceae species transcriptomes. BLASTN was used to find homologues with two different e‐values of up to 1 × 10^−05^ and 1 × 10^−30^. The resulting hits were submitted to reciprocal BLAST in NCBI and TAIR. Putative positive hits were cleaned to obtain the corresponding CDS.

Several contigs usually matched a specific gene homolog, and in turn, all variants were carefully evaluated. Gene copies were retained as such when (1) they corresponded to different contigs or (2) they corresponded to the same contig but had significant variation. For the latter, significant changes included (1) premature stop codons, (2) more than 5% differences in their CDS nucleotide sequences, and (3) alternative splice forms. However, the assessment of true copies versus allelic variation or any form of mRNA variation during and after transcription will need confirmation from genome sequencing in the future. All genes isolated in this study have been deposited in GenBank (MW356675–MW356760).

For building a comprehensive matrix for angiosperm genes as a framework for the isolated Rubiaceae genes, BLAST (Altschul et al., 1990) was implemented to search all the repositories available for plant transcriptomes such as OneKP database (https://sites.google.com/a/ualberta.ca/onekp), PhytoMetasyn (https://bioinformatics.tugraz.at/phytometasyn), and the genomes available via Phytozome (https://phytozome.jgi.doe.gov/pz/portal.html). Gene matrices also used previous data from Pabón‐Mora et al. (2014) and Ortíz Ramirez et al. (2018). All sequences used are in Appendix S2. Full‐length nucleotide sequences were compiled with Bioedit (http://www.mbio.ncsu.edu/bioedit/bioedit.html) and manually edited to exclusively keep the open reading frame for all transcripts, as many sequences include the 5′ and 3′ untranslated regions (UTRs). All sequences isolated here and those included in the analyses can be found in Appendix S2. Nucleotide sequences were then aligned using alternative algorithms. On one hand, the online version of MAFFT (http://mafft.cbrc.jp/alignment/software) (Katoh et al., 2002), with a gap open penalty of 3.0, offset value of 1.0, and all other default settings was implemented. In this case, manually refined alignments were done using Bioedit especially for large domains that were easily recognizable. Alternatively, codon‐based alignments using TranslatorX were performed (Abascal et al., 2010). Maximum likelihood (ML) phylogenetic analyses using the nucleotide sequences were performed through IQTree (Nguyen LT et al., 2015) using the CIPRES Science Gateway (Miller et al., 2010). The molecular evolution model that best fit the data was calculated using the ModelFinder tool incorporated in IQ‐TREE (Kalyaanamoorthy et al., 2017). Bootstrapping was performed with 1000 Ultra‐Fast Bootstrap replicates. Trees were observed and edited using iTOL (Interactive Tree Of Life online tool, Letunic and Bork, 2019) (https://itol.embl.de). Here, only MAFFT alignment results are shown, because alignments derived from Translator X resulted in reduced bootstrap support (BS) in phylogenetic analyses.

### Identification of protein motifs

For identifying conserved motifs and protein motifs in Rubiaceae fruit development genes, motif search analyses were done using full‐length protein sequences from *Borojoa patinoi, Cephalanthus occidentalis, Coffea arabica, Condaminea corymbosa, Galium hypocarpium, Morinda citrifolia*, and *Palicourea angustifolia*. For comparison, we also included sequences from *AGAMOUS*/*SHATTERPROOF, APETALA1/FRUITFULL, REPLUMLESS, ALCATRAZ*/*SPATULA*, and *HECATE1*/*HEC2*/*HEC3*/*INDEHISCENT* from *Arabidopsis thaliana* and some selected Solanaceae species. All sequences were permanently translated and uploaded as amino acids to the online MEME server (http://meme.nbcr.net). This suite finds in the given sequences, the most statistically significant (low E‐value) and conserved motifs across all sequences first, followed by unique motifs found only in a subset of the sequences. The e‐value of a motif is based on its log likelihood ratio, width, sites, and the size of the set.

### Developmental series and anatomy of fruits in selected species of Rubiaceae

For anatomical studies and identiflandmarks during development four species were selected. *Condaminea corymbosa* capsules *Galium hypocarpium* berries *Palicourea angustifolia* drupes *Morinda citrifolia* multiple fruits. Fruits in different developmental stages were collected in the field and immediately placed in 70% ethanol until they were dehydrated through an alcohol–Histochoice Clearing Agent (Sigma Aldrich, St Louis, MO, USA) series and embedded in Paraplast X‐tra (Leica Biosystems, Richmond, IL, USA). Samples were 10 µm with an AO Spencer 820 rotary microtome. When the embedded material was too lignified and failed to be sectioned with the microtome, sections were cut with a thin blade. Sections were stained with safranin O solution to identify lignification and presence of cuticle, and 0.5% w/v astra blue in water with tartaric acid, and mounted in Entellan (Merck, Darmstadt, Germany) and viewed and digitally imaged with a Zeiss Axioplan compound microscope equipped with a Nikon DXM1200C digital camera with ACT‐1 software (Nikon, Tokyo, Japan).

### Expression analyses using RT–PCR

For gene expression analyses, *C. corymbosa, G. hypocarpium*, and *P. angustifolia* were studied. Two different developmental stages were processed based on the previous observations in the anatomical studies. Stage 0 here is defined as the carpels in preanthetic floral buds, and Stage 1 corresponds to the first stage of fruit development documented after anthesis. Specific sizes and anatomical descriptions corresponding to these stages (preanthesis and S1) are described in the anatomical result sections. Primers were designed for all homologs found, taking special care not to use largely conserved regions for amplification (Appendix S3). Total RNA was prepared as described above from specific developmental stages. Samples were treated with DNAseI (Roche, Basel, Switzerland) and quantified with a NanoDrop 2000 spectrophotometer (Thermo Fisher, Waltham, MA, USA). Three micrograms of RNA were used as a template for cDNA synthesis (Super‐Script III RT, Invitrogen, Waltham, MA, USA) using OligodT primers. The cDNA was used undiluted for RT‐PCR. Primers were manually designed for *AP1*/*FUL, AG*/*SHP, ALC*/*SPT, HEC*, and *RPL* genes and can be found in Appendix S3. The final amplification 20‐µL reactions for the RT‐PCR included 9 µL of EconoTaq (Lucigen, Middleton, WI, USA), 6 µL of nuclease‐free water, 1 µL of BSA (bovine serum albumin; 5 mg/mL), 1 µL of Q solution (betaine, 5 μg/μL), 1 µL of forward primer (10 mm), 1 µL of reverse primer (10 mm) and 1 µL of diluted template cDNA. Expression tests were performed using a touchdown thermal cycling profile, which followed an initial denaturation step (94°C for 4 min) then (94°C for 40 s), an annealing step (10°C above real annealing temperature, for 40 s), an extension step (72°C for up to 40 s), a following step (10 cycles –1°C, each time going to step 2). This protocol was followed by a conventional thermal cycling profile with a denaturation step (94°C for 40 s), an annealing step (gene specific for 40 s), an extension step (72°C for up to 40 s) repeated for 20 amplification cycles, and a final extension (72°C for up to 10 min) and storage (4°C). *ACTIN* was used as a control. Primers pairs that resulted in no expression in the selected stages were further tested using the original mixed RNA for each species. PCR products were visually verified in a 1.5% agarose gel stained with ethidium bromide (Sigma‐Aldrich, Darmstadt, Germany), molecular weight determined, and digitally photographed using a Whatman Biometra BioDoc Analyzer.

## RESULTS

### The *APETALA1*/*FRUITFULL* (*AP1*/*FUL*) gene lineage

The *AP1*/*FUL* gene lineage evolution was reconstructed using 99 coding sequences from among all major angiosperm groups (i.e., 43 specifically from the Rubiaceae, 37 sequences from other core eudicots, eight sequences from monocots, six from basal eudicots, and five from among magnoliids, Chloranthaceae, and ANA). The molecular evolution model that best fit *AP1*/*FUL* genes was TIM + F + R4. Using *Amborella trichopoda* single‐copy *FUL* as the outgroup, the maximum likelihood (ML) phylogenetic analysis recovered the previously identified *euFULI, euFULII*, and *euAPI* clades (UFB values = 94, 100, and 100, respectively; Appendix S4).

All Rubiaceae species sampled have gene representatives from *euFULI* and *euAPI* clades (UFB = 100 and 99, respectively). In fact, most *euFULI* and *euAP1* homologs have undergone taxon‐specific duplications or are present as divergent transcript variants. Thus, *euFULI* genes are multiplied in *Condaminea corymbosa* (4 copies), *Coffea arabica* (2 copies), *Galium hypocarpium* (5 copies), *Morinda citrifolia* (3 copies), and *Palicourea angustifolia* (3 copies) (Table 1; Appendix S4). Similarly, *euAPI* genes are found duplicated in *C. corymbosa, C. occidentalis, M. citrifolia*, and *G. hypocarpium*, while four copies were found in *P. angustifolia*. All other taxa including *Coffea canephora, C. arabica*, and *Borojoa patinoi* have one *euAP1* copy. A very different trend can be seen in the *euFULII* clade, as *Cephalanthus occidentalis, Coffea arabica, C. canephora*, and *Morinda citrifolia* have one *euFULII* homolog. The exceptions are *Cinchona ledgeriana* and *Borojoa patinoi*, which have two copies. All other sampled species may have lost *euFULII* homologs altogether (Table 1; Appendix S4).

**Table 1:** Summary table of key anatomical results for fruit patterning in Rubiaceae, targeted gene copy number, and expression patterns detected in this research

					Gene copies isolated from reference transcriptomes											
	Species		Fruit and cup tissue		*AP1/FUL*			*AG/SHP*			*ALC/SPT*		*HEC/IND*			
Subfamily	Subfamily	Fruit type reported	Pericarp	Epicarp	*euFULI*	*euFULII*	*euAP1*	*AG*	*SHP*	*RPL*	*ALC*	*SPT*	*HEC1*	*HEC2*	*HEC3*	Anatomy refs
Cinchonoideae	*Cephalanthus occidentalis*	Dry indehiscent fruits (nutlets)	N/A	N/A	*CeocFUL1*	*CeocFUL2*	*CeocAP1_1*	*CeocAG1*	*CeocSHP1*		*CeocALC*				*CeocHEC3*	T.w
							*CeocAP1_2*	*CeocAG2*	*CeocSHP2*							
Ixoroideae	*Borojoa patinoi*	Fleshy fruits (berries)	N/A	N/A	*BopaFUL1*	*BopaFUL2*	*BopaAP1*	*BopaAG*	*BopaSHP*		*BopaALC*		*BopaHEC1*			T.w
						*BopaFUL3*										
	*Coffea arabica*	Fleshy fruits (drupes)	Sclerenchymatic continuous	Parenchymatic	*CoarFUL1*	*CoarFUL3*	*CoarAP1*	*CoarAG*	*CoarSHP*		*CoarALC*	*CoarSPT*	*CoarHEC1*	*CoarHEC2_1CoarHEC2_2*	*CoarHEC3*	Roth & Lindford 1971
					*CoarFUL2*											
	*Coffea canefora*	Fleshy fruits (drupes)	N/A	N/A			*CocaAP1*	*CocaAG*	*CocaSHP*		*CocaALC*	*CocaSPT*		*CocaHEC2*		
	*Condaminea corymbosa*	Dry dehiscent fruits (capsules)	Sclerenchymatic discontinuous	Parenchymatic that dries out	*CocoFUL1*		*CocoAP1_1*		*CocoSHP1*	*CocoRPL*	*CocoALC*	*CocoSPT*				T.w
					*CocoFUL2*		*CocoAP1_2*		*CocoSHP2*							
					*CocoFUL3*											
					*CocoFUL4*											
	*Cinchona ledgeriana*	Dry dehiscent fruits (capsules)	N/A	N/A		*CileFUL1*		*CileAG*			*CileALC*	*CileSPT*				
						*CileFUL2*										
Rubioideae	*Galium hypocarpium*	Fleshy fruits (berries)	Parenchymatic	Parenchymatic	*GahyFUL1*		*GahyAP1_1*	*GahyAG1*		*GahyRPL*	*GahyALC*		*GahyHEC1*	*GahyHEC2*	*GahyHEC3*	De Toni & Mariath 2011
					*GahyFUL2*		*GahyAP1_2*	*GahyAG2*								
					*GahyFUL3*											
					*GahyFUL4*											
					*GahyFUL5*											
	*Morinda citrifolia*	Multiple fleshy fruits (berries)	Parenchymatic with sclerenchyma at the apex	Parenchymatic	*MociFUL1*	*MociFUL4*	*MociAP1_1*	*MociAG1*	*MociSHP1*	*MociRPL*	*MociALC*	*MociSPT*			*MociHEC3*	T.w
					*MociFUL2*		*MociAP1_2*	*MociAG2*	*MociSHP2*							
					*MociFUL3*											
	*Palicourea angustifolia*	Fleshy fruits (drupes)	Sclerenchymatic continuous	Parenchymatic	*PaanFUL1*		*PaanAP1_1*	*PaanAG1*	*PaanSHP1*	*PaanRPL1*		*PaanSPT1*	*PaanHEC1*			T.w
					*PaanFUL2*		*PaanAP1_2*	*PaanAG2*	*PaanSHP2*	*PaanRPL2*		*PaanSPT2*				
					*PaanFUL3*		*PaanAP1_3*	*PaanAG3*	*PaanSHP3*							
							*PaanAP1_4*									

*Notes: AG, AGAMOUS; ALC, ALCATRAZ; AP1, APETALA1; FUL, FRUITFULL; HEC, HECATE; IND, INDEHISCENT; SHP, SHATTERPROOF; SPT, SPATULA; RPL, REPLUMLESS*; T.w, this work.

To assess how the *FUL* homologs in Rubiaceae have evolved compared to their counterparts in core eudicots, we evaluated a total of 39 sequences from among selected Rubiaceae, Solanaceae, and Brassicaceae sequences, using the Motif Elicitation (MEME) suite. Only proteins putatively involved in fruit development were chosen, namely, FUL proteins (from both *euFULI* and *euFULII* gene clades). Sequences of *Solanum lycopersicum* (*Sly*) and *Brunfelsia australis* (*Brau*) were included as representatives from the Solanaceae. Sequences from *Arabidopsis thaliana* (AT) where used to represent Brassicaceae homologs (Appendix S5). For this particular gene lineage, motifs were identified as described by Pabón‐Mora et al. (2014). As expected, FUL proteins have a highly conserved MADS‐box, I and K domains across all representative sequences, here recovered in motifs 1, 2, 3, 4, and 7 (Appendices S5, S6). Most changes were concentrated in the C‐terminal region, but even there some conserved motifs were identified (e.g., motifs 6, 8, 9, and 10). The FUL motif (MPPWML) is part of motif 6. Species‐specific motifs are rare, but all FULI proteins from *Galium hypocarpium* have motif 12 (RSSFBNTDTS) in the C‐terminal region. Because of the location, it may play important roles in protein–protein tetrameric interactions (Appendices S5, S6).

### The *AGAMOUS/SHATTERPROOF (AG/SHP)* gene lineage

The compiled matrix for the *AGAMOUS*/*SHATTERPROOF* (*AG*/*SHP*) lineage, included 71 sequences for the alignment (i.e., 25 from the Rubiaceae; 23 from other core eudicots, 11 from monocots, 7 from basal eudicots, and 5 sequences from among magnoliids, Chloranthaceae, and ANA). The molecular evolution model that best adjusted to the *AG*/*SHP* genes was TIM2e + R4.

The ML phylogenetic analysis of the *AG*/*SHP* gene lineage recovered the core eudicot duplication event resulting in the *AGAMOUS* (UFB = 100) and *SHATTERPROOF* clades (UFB = 99; Appendix S7). Our analysis also recovered the Brassicaceae‐specific duplication resulting in the *SHP1* and *SHP2* gene clades (UFB = 100 and 99, respectively). Rubiaceae species have retained both *AG* and *SHP* orthologues. *AG* and *SHP* genes are found as a single copy in *Borojoa patinoi, Cinchona ledgeriana, Coffea arabica, C. canephora*, and are found duplicated in all other species sampled, including *Cephalanthus occidentalis* (with 2 copies) *Galium hypocarpium* (with 2 copies), *Morinda citrifolia* (with 2 copies), and *Palicourea angustifolia* (with 3 copies). Our analyses revealed two independent losses: loss of *AG* in *Condaminea corymbosa* and loss of *SHP* in *Galium hypocarpium*. However, due to the lack of reference genome for this species such losses remain to be confirmed (Table 1; Appendix S7).

A total of 32 selected sequences including all Rubiaceae AG/SHP proteins were analyzed for conserved motifs. AG/SHP proteins are highly conserved and exhibit the MADS‐box, I, K, and C terminal domains (Appendices S8, S9). Inside the MADS box region, motifs 7 and 1 correspond to the GRGKIE and TNRQVTFCKR, respectively, two motifs characteristic of all AG/SHP proteins. The latter includes a putative phosphorylation site for the calmodulin‐dependent protein kinase. Motifs 2, 3, and 5 include most highly conserved sequences in the I and K domains. In the C‐terminal region, the AGI and AG II motifs were identified (Zhu et al., 2018). The AG motif I corresponds to motif 6 and the AG motif II corresponds to motif 9 (Appendices S8, S9).

Two new motifs were identified exclusively in Rubiaceae proteins: motif 12, upstream of the typical MADS‐box domain in all SHP homologs; motif 11, exclusively upstream of the MADS‐box domain of *Morinda citrifolia* AG proteins (Appendices S8, S9). *Morinda citrifolia* is the only species in the present study with multiple fruits. These new complete amino acid sequences were searched against the PFAM 23.0 database (http://pfam.sanger.ac.uk/) with the goal of recording any putative reported role; however, thus far, they remain uncharacterized.

### The *REPLUMLESS* (*RPL*) gene lineage

To reconstruct the RPL gene lineage evolution, we used the complete coding sequence of 34 homologs from all major angiosperm groups (i.e., 5 sequences from Rubiaceae species, 16 from other core eudicots, 3 from basal eudicots, 5 sequences from monocots, and 5 from among magnoliids, Chloranthaceae, and ANA) (Appendix S10). The molecular evolution model that best adjusted to the RPL genes was TPM3u + F + R4. Using the Amborella trichopoda single‐copy REPLUMLESS as the outgroup, the ML analysis recovered single‐copy RPL genes across the angiosperms.

As in most angiosperms, *RPL* genes are found as single copy in all Rubiaceae species sampled with a single exception; *Palicourea angustifolia* has 2 *RPL* copies (UFB = 100) (Table 1; Appendix S10). However, a detailed examination of the two sequences suggest they may be alternative transcripts, as *PaanRPL2* is shorter compared to *PaanRPL1* (Appendices S11, S12). *RPL orthologs*, however, were not recovered from *Borojoa patinoi, Cephalanthus occidentalis*, and *Coffea* spp., or *Cinchona ledgeriana*, suggesting species‐specific losses (Table 1; Appendix S10).

The *REPLUMLESS* genes belong to the HOX family, whose main characteristic is the presence of the canonical HOMEODOMAIN and BELL domain, as well as the SKY and ZIBEL motifs (Mukherjee et al., 2009; Pabón‐Mora et al., 2014; Appendices S11, S12). The MEME analysis for RPL proteins recovered those highly conserved motifs as part of motifs 1, 2, and 4 (Mukherjee et al., 2009; Pabón‐Mora et al., 2014). The SKY motif corresponds to motif 3 (SKFL) showing a substitution of the constant amino acid Y for F. Motif 11 contains the ZIBEL (VSLTL) box (Appendices S11, S12). The analysis recovered motif 10 as mostly exclusive to Rubiaceae RPL proteins. Finally, motif 6 is absent in *Condaminea corymbosa*, the dried fruited species. None of the latter two motifs have any recorded function.

### The *ALCATRAZ*/*SPATULA* (*ALC*/*SPT*) gene lineage

The alignment for the *ALC*/*SPT* gene lineage includes 56 sequences from among all major seed plant groups (i.e., 14 sequences from Rubiaceae species, 27 from other eudicots, 6 from monocots, 3 from basal eudicots, and 5 from among magnoliids, Chloranthaceae, and ANA; Appendix S13). The molecular evolution model that best adjusted to the *ALC*/*SPT* genes was TPMuF+R4. The topology of this tree recovered the core eudicot duplication resulting in the *ALC* (UFB = 93) and *SPT* (UBF = 93) clades (Pabón‐Mora et al., 2014; Zumajo‐Cardona et al., 2017; Appendix S13). As a result, most core eudicots, including members of the Rubiaceae have *ALC* and *SPT* orthologs. In addition, species‐specific duplications of *SPT* genes were detected in *Palicourea angustifolia* (Rubiaceae) and *Brassica rapa* (Brassicaceae). Possible losses in Rubiaceae were identified for *ALC* orthologs in *Palicourea angustifolia* and *SPT* orthologs in *Borojoa patinoi, Cephalanthus occidentalis*, and *Galium hypocarpium* (Table 1; Appendix S13). However, as explained above, confirmation of these results is required upon genome availability for these species.

A total of 21 ALC/SPT proteins were analyzed using the MEME suite. The bHLH (basic, Helix1, Loop and Helix 2; Groszmann et al., 2011) domain corresponds to motifs 1 and 3. The bipartite NLS segment is part of motif 3 in our analysis. Both the bHLH and the NLS segment are present in all ALC/SPT proteins evaluated as expected (Appendices S14, S15). Finally, the acidic domain corresponds to motif 8, and it is only present in the majority of the SPT proteins while lacking in the ALC proteins.

Different exclusive motifs have been fixed independently in Rubiaceae ALC and SPT proteins (Appendices S14, S15). Motifs 5, 6, and 10 are only present in Rubiaceae ALC proteins. On the other hand, motifs 7 and 9 are found in most Rubiaceae SPT proteins. Also, motif 11 is present in a few SPT Rubiaceae sequences at the beginning of the bHLH domain (Appendices S14, S15).

### The *HECATE*/*INDEHISCENT* (*HEC*/*IND*) gene lineage

The phylogenetic reconstruction of the *HEC1*/*2*/*3*/*IND* gene lineage was generated using 72 sequences (i.e., 13 from the Rubiaceae, 35 from other core eudicots, 10 from monocots, 5 from species of basal eudicots, and 9 from magnoliids, Chloranthaceae, and ANA; Appendix S16). The ML analysis recovered an early duplication event for all angiosperms resulting in the *HEC1*/*2* (UFB = 88) and the *HEC3*/*IND* (UFB = 78) clades (Pfannebecker et al., 2017). Our analysis also recovered local duplications in Brassicaceae resulting in the *HEC1* (UFB = 100) and *HEC2* (UFB = 100) clades and in the *HEC3* and *IND* clades (UFB = 97). Sampling within Rubiaceae only recovered *HEC1* orthologs from *Borojoa patinoi, Coffea arabica, Galium hypocarpium*, and *Palicourea angustifolia; HEC2* orthologs from the two species of *Coffea* and *Galium hypocarpium*, and finally, *HEC3* orthologs from *Cephalanthus occidentalis, Coffea arabica, Galium hypocarpium, Morinda citrifolia*, and *Palicourea angustifolia*. Thus, only *G. hypocarpium* and *Coffea arabica* have all three expected *HEC* copies, while from all other Rubiaceae sampled, only one or two *HEC* homologs were recovered (Table 1; Appendix S16).

A total of 24 selected HEC/IND proteins were analyzed in the search of conserved motifs using the MEME suite (Appendices S17, S18). The highly conserved bHLH domain corresponds to motifs 1, 2, and 6 (Appendices S17, S18). Motifs were identified as described by Gremski et al. (2007) and Groszmann et al. (2008). Exclusive domains found for all Rubiaceae proteins were not identified. However, motifs 5 and 11 are specific to a few HEC1 and HEC3 Rubiaceae proteins. Similarly, motifs 4, 7, and 12 are exclusive of a few HEC2 Rubiaceae proteins (Appendices S17, S18).

### Floral and fruit anatomy in *Condaminea corymbosa*

**Figure 2:**
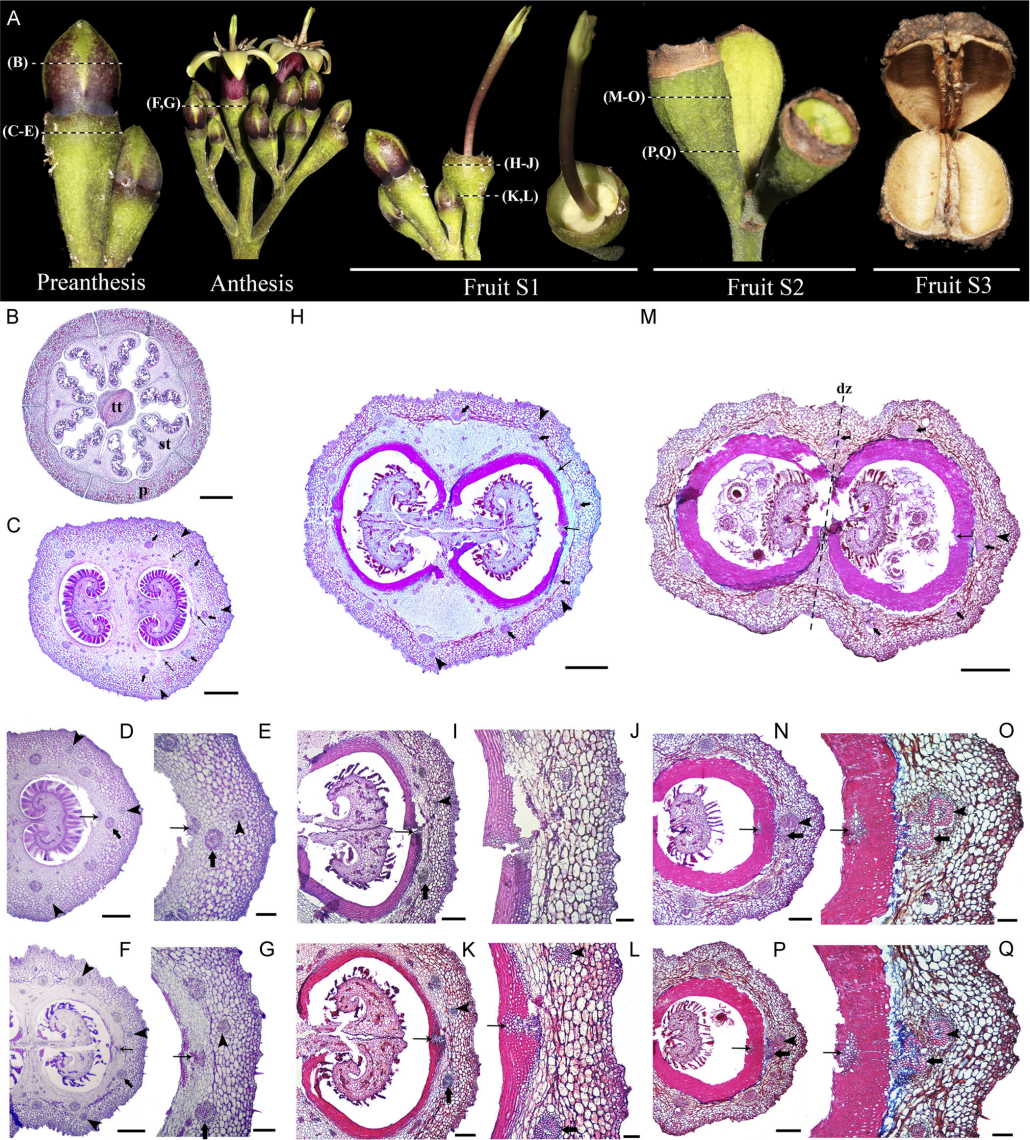
(A) Developmental stages of *Condaminea corymbosa* flower to fruit transition. (B–G) Floral bud cross sections. (H–L) Cross sections of the fruit at Stage 1 (S1). (M–Q) Cross sections of the fruit at Stage 2 (S2). dz, dehiscence zone; p, petal; st, stamen; tt; transmitting tract tissue; arrowhead, outermost vascular ring likely irrigating the sepals; thick arrows, middle vascular ring irrigating petals and stamens; thin arrows, innermost vascular ring irrigating the carpels. Scale bars = 10 µm in J, L; 20 µm in E, G, O, Q; 50 µm in D, F, I, K, N, P; 0.5 mm in M; 1 mm in B, C, H

Condaminea corymbosa flowers are pentamerous with a tubular calyx formed by five sepals, five connate petals, five adnate stamens, and a bicarpellate gynoecium within a concave floral cup (Figure 2A–C). The inferior ovary has two locules with numerous ovules arranged in an axillar placentation in each carpel (Figure 2C–G).

Floral vascularization of the inferior ovary/cup fusion shows three concentric rings of vascular bundles, hereafter referred to as the outer, middle, and inner rings. The outer ring has a total of six vascular traces, three in each locule. The middle ring has 10 vascular traces, five in each locule. The innermost traces are scattered in the floral cup reaching 30 smaller bundles (Figure 2C, D, H). In pre‐anthesis, the ovary wall and the floral cup form a continuous tissue of 20–22 cell layers (Figure 2A, C). The epidermis is formed by small isodiametric cells, sometimes interrupted by stomata or trichomes covered by cuticular wax (Figure 2C). The remaining cell layers vary dramatically depending on the level at which the cross section is made in the flower. Toward the apex of the ovary/cup, the cells surrounding the inner and medial rings of vascular tissue (ca. 10 layers) remain mostly parenchymatic, while collenchyma occupies the 12–14 layers from the outer vascular ring until the epidermis (Figure 2C, D). Moreover, collenchymatic cells closer to the epidermis are smaller than those closer to the vascular bundles (Figure 2D, E). Conversely, toward the base of the ovary/cup the distinction between the inner parenchyma and outer collenchyma is more evident, as some cells in between the two tissues appear crushed. Cellular changes are likely due to cell thickening in the inner collenchyma cells and the lack of periclinal cell division (Figure 2F, G). However, the most striking difference at this level is the beginning of lignification in the innermost epidermis and the 2–3 layers adjacent to it, as well as in the proximal corners of the placenta (Figure 2F, G). Sclerenchyma is only beginning to differentiate, and it does not form a continuous layer through the pericarp at this time (Figure 2F, G).

Post‐anthesis marks the beginning of fruit development after pollination takes place. Stage 1 (S1) for fruit development is here recognized as a fruit that reaches ca. 5 mm (Figure 2A, H–L). The fruit/cup continuum at S1 changes dramatically, and three distinct tissues are readily seen across the ca. 28 layers in both the apex (Figure 2I, J) and the base (Figure 2K, L). From the inside out, the first 8–10 layers are now completely lignified (Figure 2I–L). The two medial innermost vascular bundles are embedded in the lignified tissue, but most of the peripheral inner small vascular traces do not integrate into the sclerenchymatic layers and remain surrounded by parenchymatic cells (Figure 2H). Parenchymatic tissue occupies the next ca. 6–8 cell layers flanked on the outside by the middle ring of vascular bundles (Figure 2I–L). Next, ca. 10 layers of collenchyma cells differentiate outside of the middle vascular ring and surrounding the outer ring of bundles (Figure 2I, L). Finally, the epidermis is found, covered by a thick cuticle (Figure 2I–L). The main difference between the apex and the base of the elongating fruit/cup continuum is the degree of lignification, and at the top, some of the inner cell lumen still remains (Figure 2I, J), while in the latter, most cells forming the lignified tissue lack lumen (Figure 2K, L). At S1, the lignified proximal corners of the placenta fuse with the lignifying layers of the pericarp; however, the placenta attachment point does not lignify, nor does the septum, leaving parenchymatic tissue in between the two locules (Figure 2H–L).

In Stage 2 (S2) of fruit development, the fruit/cup retains ca. 28 layers, suggesting that periclinal cell division no longer occurs, but anticlinal cell division continues as the fruit reaches ca. 1 cm (Figure 2A). At Stage 2, up to ca. 13 lignified cell layers have formed (Figure 2M–Q). Smaller traces corresponding to the inner ring are no longer detected (Figure 2M–Q). As the fruit matures, the parenchymatic tissue is reduced to ca. 5 layers, adjacent to 9 or 10 layers of collenchymatous tissue (Figure 2N–Q). There are no major differences between the anatomy of the apex and the base of the fruit/cup.

The fruit reaches ca. 1.5 cm at Stage 3 (S3), and lignification of the pericarp has completed (Figure 2A). The final result is a dehiscent, dry fruit (capsule) with a septicidal opening for seed dispersal (Figure 2A).

### Floral and fruit anatomy in *Galium hypocarpium*

**Figure 3:**
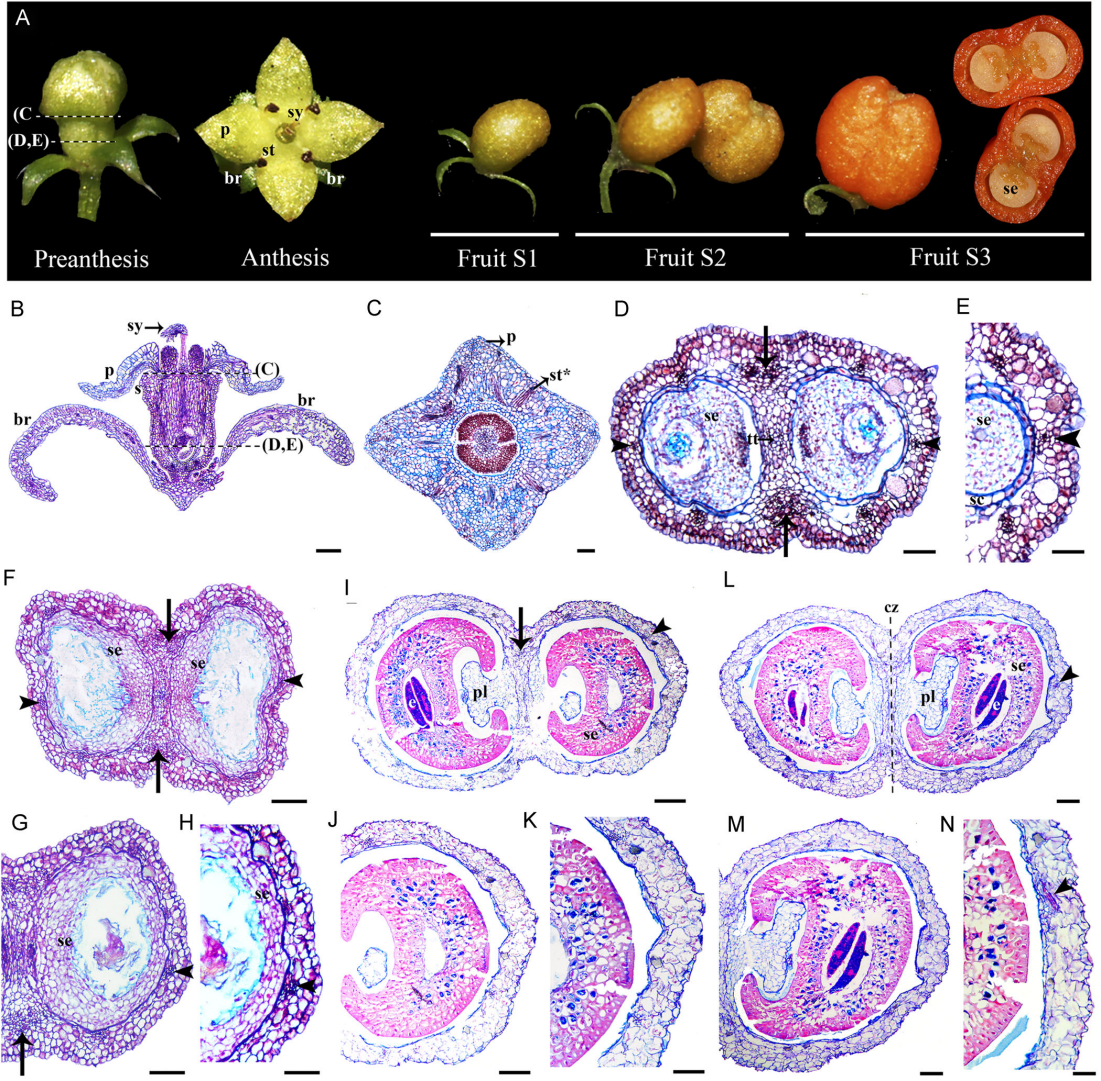
(A) Developmental stages of *Galium hypocarpium* flower to fruit transition. (B) Floral bud longitudinal section. (C–E) Cross sections at two levels of the floral bud, at the level of petal and stamen insertion (C) and at the ovary/cup level (D, E). (F–H) Cross sections of the fruit at Stage 1 (S1). (I–K) Cross sections of the fruit at Stage 2 (S2). (L–N) Cross sections of the fruit at stage 3 (S3). br, bract; cz, constriction zone; e, embryo; p, petal; s, sepal; sc, seed coat; se, seed; st∗, stamen vascular trace; sy, style; arrowhead, single vascular ring; arrows, medial bundles likely irrigating the disk. Scale bars = 50 µm in B–E; 100 µm in F–H; 200 µm in I–K; 250 µm in L–N

Flowers of *Galium hypocarpium* consist of four connate sepals, four petals, four stamens alternating to the petals, and two carpels with inferior ovaries (Figure 3A, B). A nectar disk at the level of petal and stamen insertion surrounds the style in the flowers (Figure 3C). A single ovule is present in each carpel (Figure 3B, D).

Floral vascularization shows six vascular bundles irrigating the floral cup/ovary continuum (Figure 3D). They will supply vascular traces for all floral organs at the top levels. In addition, there are two massive medial bundles to irrigate the disk (Figure 3D). Pre‐anthesis, the ovary wall and the floral cup form a continuous tissue of ca. 5 or 6 layers (Figure 3D, E). The ovary wall/cup has outer idioblasts rich parenchyma cells and inner smaller parenchyma cells (Figure 3E). The epidermis is formed of smaller rectangular cells sometimes interrupted by papillary cells and trichomes, both with a thick cuticle (Figure 3D).

We were not able to directly trace the timing of fertilization and fruit initiation. Nevertheless, post‐anthesis, when fruits reach ca. 2 mm in size, is here recognized as fruit development Stage 1 (S1) (Figure 3A). The fruit/cup continuum tissues at S1, have homogenous large parenchymatic cells with sparse idioblasts (Figure 3F–H). No periclinal cell division is evident concomitant with fruit formation (Figure 3F) because the same number of cell layers are present compared to earlier stages. Vascular traces from the fruit/cup are in direct contact with the seed coat, which begins to separate and isolate itself from the fruit wall (Figure 3F–H). The endosperm tissue is massive inside the seed (Figure 3F), and the embryo has begun its differentiation (Figure 3G, H).

At S2 (ca. 4 mm fruit size), the fruit/cup continuum undergoes extreme vacuolization (Figure. 3I–K) as parenchymatic cells enlarge and sometimes merge with each other. The placenta remains fully parenchymatous (Figure 3I). The only layer that keeps its integrity is the epidermis (Figure 3J, K). The seeds undergo heavy lignification as the embryo grows (Figure 3I–K). The vascular bundles are retained as small units within the pericarp (Figure 3I). The same fruit/cup tissues are retained as the fruit enlarges and reaches Stage 3 (S3) (Figure 3L–N). At this stage, a constricted zone begins to form in between the two carpels (Figure 3L). However, the two units never separate as in other schizocarps reported for *Gallium* species (De Toni and Mariath, 2011).

### Floral and fruit anatomy in *Palicourea angustifolia*

**Figure 4:**
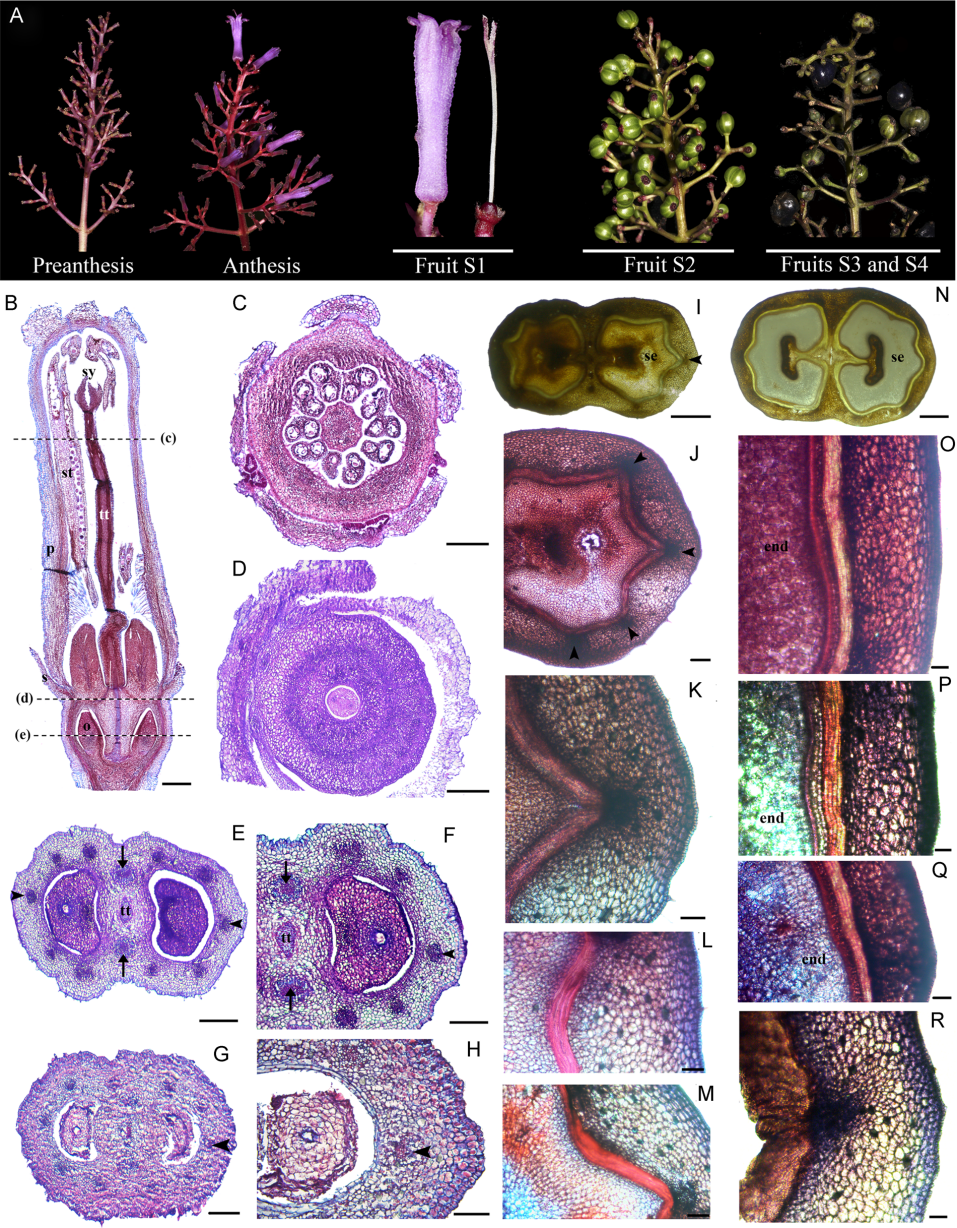
(A) Developmental stages of *Palicourea angustifolia* flower to fruit transition. (B) Floral bud longitudinal section. (C–E) Cross sections in the three levels (floral organs, disk, and ovary) pointed in (B). (F) Detail of the floral cup/ovary level in (E). (G, H) Cross sections of the fruit at Stage 1 (S1). (I–M) Cross sections of the fruit at Stage 2 (S2). (N–R) Cross sections of the fruit at Stage 3 (S3). o, ovule; p, petal; s, sepal; se, seed; st, stamen; sy, style; tt, transmitting tract tissue; arrowhead, single vascular ring; arrows, medial bundles likely irrigating the disk. Scale bars = 20 µm in F, H, K–M, O–R; 50 µm in C, D, E, G; 0.5 mm in B; 1 mm in I, N

Flowers of *Palicourea angustifolia*, consist of five connate sepals, five connate petals, five adnate stamens, and a bicarpellate gynoecium (Figure 4A–D). The gynoecium differentiates into an apical bifurcated stigma, a thick style with well‐developed transmitting tissue, and a massive solid inferior ovary (Figure 4A–C). The ovary apex forms a continuous tissue arranged as a ring around the style, named the “disk” (Figure 4D; Igersheim et al., 1994). A single, basally attached ovule is present in each carpel (Figure 4B, E, F).

Ten vascular bundles irrigate the floral cup and the inferior ovary, forming a single ring (Figure 4B, E, F). They supply the vascular traces for all floral organs. In addition, two medial lateral bundles flank the transmitting tissue and supply the disk (Figure 4B). The two main ovary traces run along the style and the stigmas (Figure 4B–D).

Pre‐anthesis, the ovary wall and the floral cup form a continuous tissue (Figure 4B, E, F). However, the vasculature divides the cells in two different tissues. Inside the vascular bundles are 6–8 cell layers of small, nonvacuolated, tangentially elongated cells. Surrounding the vascular bundles and up to the epidermis are 10–12 layers of largely vacuolated, isodiametrical cells (Figure 4E, F). Frequently, idioblasts can be distinguished among them, perhaps with tannins. The epidermis is formed of rectangular cells sometimes interrupted by papillary cells, both with a thick cuticle (Figure 4E, F).

Although we were not able to directly trace fertilization, only after anthesis is the pollen tube free to enter the transmitting tissue and reach the ovules. Thus, fruits reaching ca. 3 mm in size in post‐anthesis are here recognized as fruit development Stage 1 (S1) (Figure 4A). The fruit/cup continuum tissues at S1 can be distinguished from the inside out into 3–4 inner layers with tangentially elongated cells, followed by ca. 12 layers of small isodiametric cells inner to the vascular bundles. Surrounding the bundles into the epidermis, the tissue consists of expanded cells that are largely vacuolated, and idioblasts are more frequent. Epidermal cells continue to be radially elongated (Figure 4G, H).

As the seeds mature and expand to fill in the locules during Stage 2 (S2), anticlinal and periclinal cell division occurs in the surrounding fruit/cup (Figure 4A, I–M). At S2 (ca. 5 mm fruit size), the five vascular bundles in each locule become major positional landmarks for tissue transformation. At this stage, the 3–4 inner layers inside the vascular bundles become lignified and form a continuous endocarp in each of the locules. The lignification reaches into the placenta. The rest of the cell layers in the fruit/cup can be distinguished into ca. 17 layers of parenchymatous cells that surround the vascular bundles, smaller in the inside and larger on the outside, as well as 3–5 layers of collenchymatous cells differentiated in the proximity of the epidermis. At this stage, the endosperm is surrounded by parenchymatous tissue in the testa, which remains in close contact with the lignified endocarp (Figure 4I–M).

At Stage 3 (S3) when the fruit reaches ca. 7 mm, lignification of the testa marks the most important change with respect to S2 (Figure 4A). In the pericarp, the lignified endocarp completes its development and reaches the placenta, completely surrounding each seed (Figure 4N). Because the seed remains in close contact with the pericarp, the lignified endotesta accompanies the outer, still parenchymatous exotesta.

The latter is in turn appressed to the fully lignified endocarp. The vascular bundles remain embedded in parenchymatous cells that have begun expansion and have stopped cell division. The 3–4 outer collenchymatous cell layers underneath the epidermis still remain (Figure 4N–R).

### Floral and fruit anatomy in *Morinda citrifolia*

**Figure 5:**
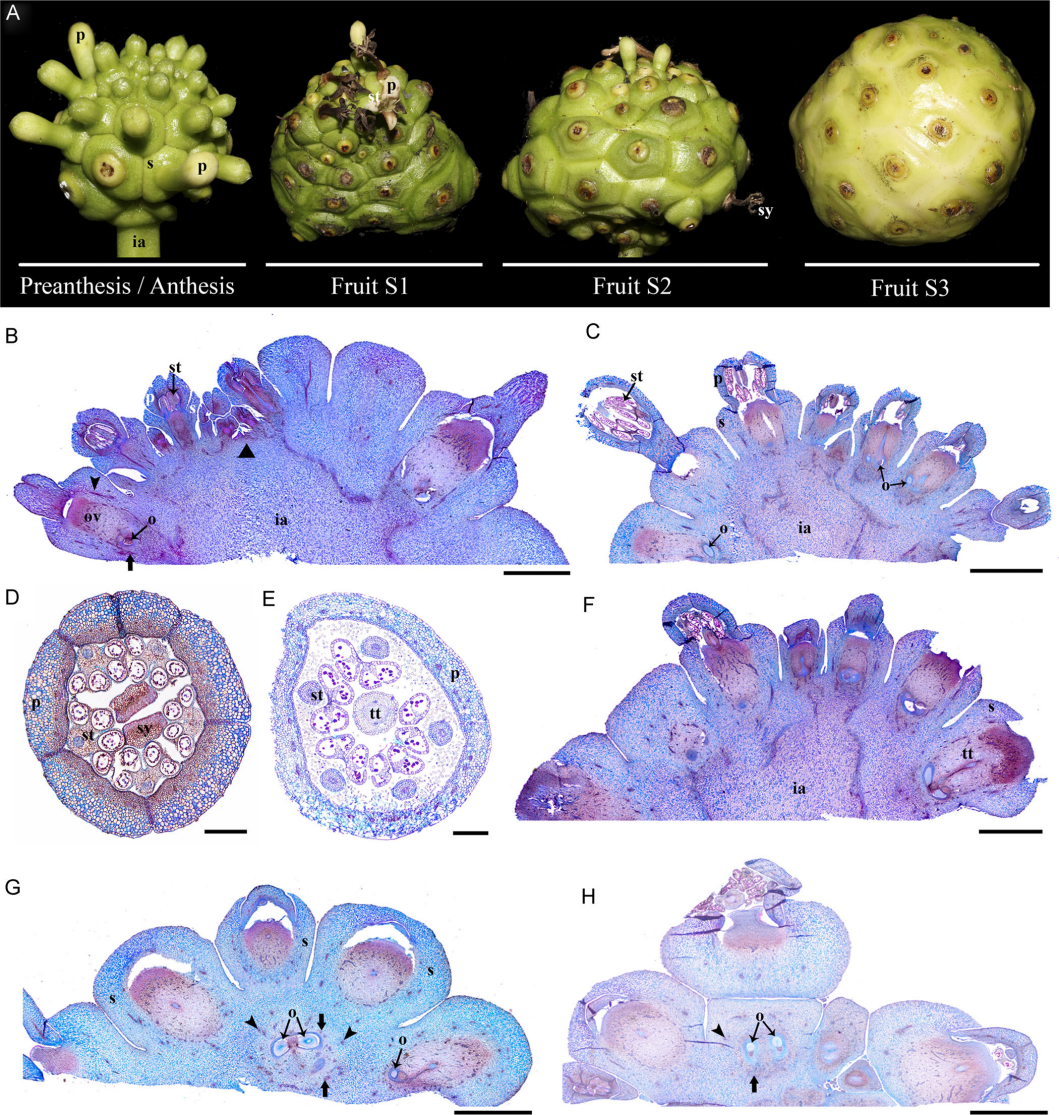
(A) Developmental stages of *Morinda citrifolia* flower to fruit transition. (B, C) Longitudinal sections of a young inflorescence predominantly with preanthetic flowers. (D, E) Cross sections of young (D) and old (E) preanthetic flowers, dissected from the inflorescence. (F, G) Longitudinal sections of infructescence at Stage 1 (S1). ia, inflorescence apex; o, ovule; p, petal; s, sepal; st, stamen; sy, style; tt, transmitting tract tissue. arrowhead, single vascular ring; arrows, medial bundles likely irrigating the disk. Scale bars = 2 mm in B, C, F–H; 150 µm in D; 250 µm in E

Flowers of *Morinda citrifolia* are pentamerous, with thick sepals forming a ring, alternating petals and stamens, and a massive bicarpellate gynoecium with an inferior ovary and a protruding style and stigma (Figure 5A–F). The gynoecium locules are extremely reduced, and the connecting transmitting tissue grows surrounded by a massive ring disk, similar to that of *P. angustifolia* (Figure 5B). As flowers grow, the bracts and the massive sepals from neighboring flowers come into closer contact. Petals, which start as independent primordia, fuse during flower development (Figure 5D, E). The base of the filaments is surrounded by numerous trichomes (Figure 5E). The style is formed by a massive transmitting tissue, and the stigmas can be or bi‐ or tri‐lobed (Figure 5D, E).

Pre‐ and post‐anthesis, the ovary wall and the floral cup form a continuous tissue (Figure 5E–H). However, different vascular bundles from those in the inflorescence axis irrigate the sepals and the ovary (Figure 5B, C, G, H). Fruit development begins after anthesis. However, we were not able to directly trace fertilization at this stage, where the pollen tube is free to enter the transmitting tissue and reach the ovules. Because flowers developed acropetally, fruits also begin maturation in the same direction. In turn, the infructescence is actively thickening at the base and the apex will often have some anthetic flowers (Figure 5A). Stage 1 (S1) fruit development is the stage when the last flowers at the apex of the infructescence are finishing anthesis (Figure 5A). The infructescence reaches ca. 2 cm. Also at this stage, sepals and ovaries are thickening simultaneously during fruit development (Figure 5F–H). The sepal‐ and the ovary‐derived tissues are always separated by the vascular traces (Figure 5F–H). While sepal‐derived tissues remain collenchymatic, major changes occur in the ovary‐derived tissues. In particular, the apical disk surrounding the transmitting tract enlarges and protrudes above the sepals, and sclerenchymatic tissue is accumulating as the fruits and the infructescence matures (Figure 5A, F–H). In this same stage, the seeds mature and expand to fill in the locules (Figure 5G, H).

The next stages of fruit development, S2 and S3, were hard to evaluate due to their large size (3 and 4 cm, respectively; Figure 5A). However, in handmade sections, it was evident that tissues from the inflorescence axis and the flowers completely merge, but the vascular bundles and some tissue differentiation are retained in the sepal‐ versus the ovary‐derived tissues. At S3, ripening begins and is accompanied with a shift to the yellowing and softening of all tissues, except for the remnants of the sclerenchymatic ovary disk.

### Gene expression analyses of candidate fruit development genes in three selected species through RT–PCR

**Figure 6:**
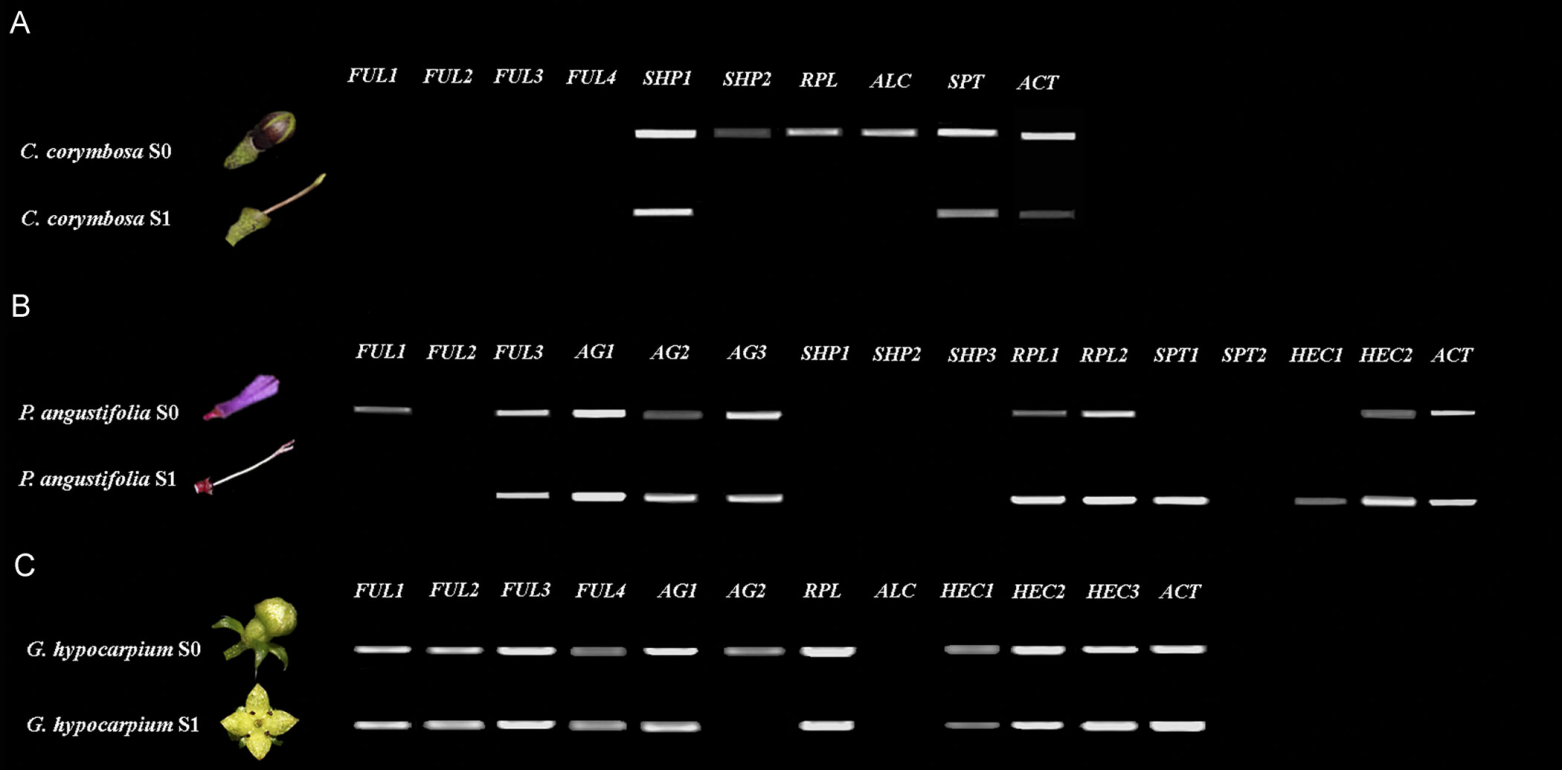
Expression analyses of fruit development genes in *Condaminea corymbosa* (with capsules), *Galium hypocarpium* (with berries), and *Palicourea angustifolia* (with drupes)

Toward understanding the roles of fruit development homologs (*AG*/*SHP, FUL, RPL, ALC, SPT* and *HEC1*/*2*/*3*) in Rubiaceae, three species were studied further. Fruits of *Condaminea corymbosa* (capsules), *Palicourea angustifolia* (drupes), and *Galium hypocarpium* (fleshy fruits), were dissected in stages S0 (carpels of pre‐anthetic floral buds) and S1 (early fruit development) (Figure 6). Gene expression for all copies found were evaluated using the actin gene as a positive control (Figure 6).

The *euFULI* genes were expressed differently in the three species. Most of the *FUL* copies from *P. angustifolia* and *G. hypocarpium* were expressed in the carpels pre‐anthesis, but only expression of *GahyFUL1‐4* and *PaanFUL3* was maintained during early fruit development. Surprisingly, the *C. corymbosa FUL* homologs were not expressed in S0 or S1. *AG* homologs were only identified in *P. angustifolia* and *G. hypocarpium*, and all copies found were broadly expressed in S0 and S1 with the exception of *GahyAG2*, that was turned off in young fruits. On the other hand, *SHP* orthologs were only identified in *C. corymbosa* and *P. angustifolia*, but were only active at S0 and S1 (*CocoSHP1* at least) in the dry fruits of *C. corymbosa*.

The expression of *RPL* genes in the three species was very different. The *P. angustifolia* and *G. hypocarpium RPL* homologs were expressed in carpels and young fruits, while the *RPL* homolog from *C. corymbosa* was only active during carpel development. *ALC* orthologs were only recovered from *C. corymbosa* and *G. hypocarpium*, but only *CocoALC* was active during carpel development. *SPT* homologues were recovered from *C. corymbosa* and *P. angustifolia* and were active in both species during carpel and early fruit development, with the exception of *PaanSPT2* whose expression was undetected in carpels or young fruits.

Importantly, *HEC* homologues were only found in *P. angustifolia* and *G. hypocarpium*. While all *HEC* homologues in *G. hypocarpium* and *PaanHEC2* were expressed at S0 and S1, expression of *PaanHEC1* was low at S1 (Figure 6). For amplifying all genes isolated from the transcriptomes, mixed cDNA was used to confirm the validity of all contigs including those that were not active in the two stages tested (Appendix S19).

## DISCUSSION

### Fruit types in the Rubiaceae and modifications in the floral cup

With the goal of evaluating the axial versus appendicular nature of the floral cup in Rubiaceae, we paid special attention to vasculature traces. To date, on the basis of the presence of 2–3 concentric rings of vasculature surrounding the locules, several Rubiaceae ovaries have been assigned to the most frequently occurring appendicular type floral cup (Fukuoka, 1980; Igersheim, 1993; Igersheim et al., 1994; Dessein et al., 2001). In fact, the outermost bundles are thought to correspond to the sepal‐irrigating traces; the middle bundles to petal and stamen traces, and the innermost bundles to the ovary traces (Fukuoka, 1980). Our observations, at least in *Condaminea corymbosa* and *Morinda citrifolia*, show 2–3 vascular rings supporting the appendicular origin of the floral cup. The presence of multiple vascular rings contrasts with the reduction to a single ring of vasculature in *Galium hypocarpium* and *Palicourea angustifolia*, which could be indicative of independent origins with the axial nature of the floral cup or, more likely, a result of extreme reduction in floral size.

Less is known about the transformation of the floral cup tissue during fruit maturation and the effect it has on the final mechanisms used for seed dispersal in different taxa. For example, in Melastomataceae, the presence of a hypanthium is associated with the presence of fleshy indehiscent fruits, whereas capsules form mostly from superior ovaries (Clausing et al., 2000; Cortez and Carmello‐Guerreiro, 2008). However, in Rubiaceae, fleshy, drupaceous, and capsular fruits can occur from inferior ovaries (Figures 2, 3, 4, 5). The only evolutionary trend identified so far in Rubiaceae is the recurrent, independent acquisition of fully fleshy fruits in different groups from a dry‐fruited ancestor (Eriksson and Bremer, 1991; Razafimandimbison et al., 2012). However, it is possible that the fleshiness optimized in most phylogenetic analyses comes from the extracarpellary floral‐derived tissues persisting during fruit maturation of an inferior ovary.

The floral‐cup‐derived tissues have recently been integrated into the histogenetic terminology coined for the pericarp (i.e., endocarp, mesocarp, and exocarp) and collectively are called the epicarp (Bobrov and Romanov, 2019). Nevertheless, the practical identification of the limits between the floral cup and the gynoecium‐derived tissues in Rubiaceae seems to be complicated by the variation in the number of concentrical rings of vasculature (1–3) and the anatomical changes in between. Small flowers, like those of *G. hypocarpium* and *P. angustifolia* tend to have a single vascular ring, which invariably nourishes the ovary tissue. This ring then splits above the ovary level to supply the independent portions of the floral organs (Figures 3 and 4). Larger flowers, like those of *C. corymbosa* and *M. citrifolia*, have three vascular rings. The innermost supplies the ovary, the middle ring irrigates the stamens and petals, and frequently, and the outer ring supplies the sepals (Figures 2 and 5).

Our data suggests that most tissues present outside the innermost vascular ring correspond to the epicarp (Figures 2, 3, 4, 5). If so, the epicarp remains fleshy and can thicken, undergo cell expansion and ripening, independent of the transformations of the pericarp. The pericarp can remain parenchymatic, like in *G. hypocarpium* (Figure 3), transform into a continuous or discontinuous sclerenchymatic tissue, like in the case of *P. angustifolia* (Figure 4), and *C. corymbosa* (Figure 2), respectively, or alternatively, have a combination of collenchymatic and sclerenchymatic tissues, like in *M. citrifolia* (Figure 5). Changes in the pericarp and epicarp control differences in the seed strategy dispersal. For instance, in *C. corymbosa*, the epicarp dries out, and the placenta and the seeds are released at maturity. Conversely, in *G. hypocarpium, M. citrifolia*, and *P. angustifolia*, the fleshy epicarp remains until maturity and thickens, protecting the seeds.

### Genetic complement of the fruit patterning genes in the Rubiaceae

As the fourth largest plant family in terms of species number, the Rubiaceae have one of the most diverse repertoires of fruit types. However, the genetic mechanisms responsible for the diversity of anatomical changes during the carpel to fruit transformation are poorly studied. A first step for comparative developmental studies of fruit development in Rubiaceae is the isolation and preliminary analyses of protein sequences and expression patterns of the main gene families controlling fruit histogenesis in *Arabidopsis*. In this study, we isolated homologs of *AP1*/*FUL, AG*/*SHP, RPL, SPT*/*ALC*, and *HEC*/*IND* from seven species spanning the three subfamilies: Cinchonoideae, Ixoroideae, and Rubioideae. Here we concentrate on discussing their evolution and expression only and will not emphasize the MEME analyses, because the motifs found are mostly those that define the different gene families. In the results, we reported the protein motifs that in fact characterize Rubiaceae proteins or those that appear to be synapomorphies or autapomorphies for some species, but we will not discuss them further because their functions are still unknown. Future experiments are needed to functionally characterize these exclusive motifs in further detail and analyze their contribution to protein function in different fruit types.

### *AP1*/*FUL* gene homologs in Rubiaceae

*FRUITFULL* (*FUL*) belongs to the *APETALA1* (*AP1*)/*FRUITFULL* MADS‐box gene lineage. *FUL* is essential for the normal growth and differentiation of valve cells while repressing the expression of *SHP, IND*, and *ALC*, which are involved in the identity of the valve margin (Ferrándiz et al., 2000). Its closest homologs in *Arabidopsis, AGL79* and *AP1*/*CAL*, are specialized for root development and perianth identity, respectively (Coen and Meyerowitz, 1991; Parenicová et al., 2003). Our phylogenetic analyses recovered the three major core eudicot clades of the *AP1*/*FUL* gene lineages, namely, the *euAP1, euFULI* and *euFULII* genes (Appendix S4; Pabón‐Mora et al., 2014).

All Rubiaceae species sampled have one to two *AP1* copies, with the exception of *Palicourea angustifolia* with four copies. All of them are species‐specific duplicates. The occurrence of *AP1* as single copy is a common trend found in other core eudicots, with the exception of the Brassicaceae with 2–4 copies in two clades, namely, *AP1* and *CAL*, as a result of a duplication event before the diversification of the family (Litt and Irish, 2003). *FRUITFULL* genes are by far more diverse, when compared to *euAP1* genes (Appendix S4). Our analyses resulted in a larger number of *euFULI* homologs (17 genes) isolated when compared to the *euFULII* homologs (5 genes). Such diversity of *FULI* genes is particularly noticeable in *Galium hypocarpium, Morinda citrifolia*, and *Palicourea angustifolia*, all of which are members of the Rubioideae (Table 1; Appendix S4). There is no obvious relation between the increase in copy number and a particular fruit feature, given that they range from berries (*Galium hypocarpium*), to multiple and fleshy (*Morinda citrifolia*), to drupes (*Palicourea angustifolia*).

Expression analyses by RT‐PCR was evaluated only for *euFULI* genes in pre‐ and post‐anthetic carpels during early fruit development. As expected, most of them are active in the two stages in *G. hypocarpium* and *P. angustifolia*. The exception is the lack of expression of all *FUL* homologs in *C. corymbosa* during S0 and S1, which was unexpected because these genes play key roles in carpel and fruit development. This case is worth studying carefully in the future, because this species lacks *euFULII* homologs that could be fulfilling the fruit patterning roles at these early stages. One aspect worth highlighting is the slightly different expression patterns recorded for alternate splice forms or different *euFULI* loci in *P. angustifolia*, which suggest they are not fully redundant (Figure 6). The expression of the *euFULI* orthologs in the Rubiaceae is consistent with their putative roles in proper cell division during carpel and fruit wall development in most core eudicots studied so far (Müller et al., 2001; Jaakola et al., 2010; Bemer et al., 2012). However, spatiotemporal gene expression analyses are needed to identify whether the roles of *FUL* genes in Rubiaceae are restricted to ovary‐derived tissues or have expanded their expression domains and functions to extracarpellary tissues.

### *AG/SHP* **gene homologs in Rubiaceae**

*AGAMOUS* and *SHATTERPOOF* are the MADS‐box founding members of the *AG*/*SHP* gene lineage. *AG* is probably the most essential gene in plant sexual reproduction, since it controls stamen and carpel identity and floral meristem determinacy (Bowman et al., 1989, 1991; Coen and Meyerowitz, 1991). On the other hand, the two *SHATTERPROOF* genes (*SHP1* and *SHP2*) in *Arabidopsis* specify valve margin identity and the formation of the dehiscence zone during fruit development (Liljegren et al., 2000). *AG* and *SHP* homologs are present in all core eudicots because their origin maps to a duplication event before the diversification of this large plant group (Kramer et al., 2004; Appendix S7). Consistent with this pattern, the Rubiaceae species sampled have at least one (and up to two) representative gene in each clade (Appendix S7; Table 1). The exceptions are *Condaminea corymbosa* for which no *AG* homologs were found and *Galium hypocarpium* for which no *SHP* genes could be isolated.

RT‐PCR expression patterns detected for *AG*/*SHP* homologs show a degree of functional compensation. While no *AG* genes were detected in *C. corymbosa*, the three *AG* copies isolated from *P. angustifolia* and the two copies of *AG* found in *G. hypocarpium* are broadly expressed and active in the carpel during the transition to fruit development (Figure 6). Conversely, although *SHP* genes are not expressed in any of the carpel to fruit stages sampled in *P. angustifolia*, the two *SHP* paralogs in *C. corymbosa* are expressed in the carpel, and at least *CocoSHP1* remains active until fruit development (Figure 6). These results raise the possibility that *SHP* orthologs can compensate for fundamental roles in stamen and carpel identity, at least in *C. corymbosa*. This possibility is highly likely because *SHP* and *AG* and their close paralogs *STK* play redundant roles in ovule development and have common functions in carpel identity in *Arabidopsis* (Pinyopich et al., 2003). Similarly, in other core eudicots including *Nicotiana benthamiana* and *Petunia hybrida*, both *AG* and *SHP* orthologs have redundant stamen and carpel identity roles (Fourquin and Ferrándiz, 2012; Heijmans et al., 2012).

The idea of functional compensation between lineage members during evolutionary time is supported by data from *Antirrhinum majus*, where *PLENA*, the *SHP* ortholog, plays the same roles as *AG* in *Arabidopsis* (Davies et al., 1999). However, functional compensation in the opposite direction, where *AG* orthologs can control the differentiation of the valve margins and the formation of the dehiscence zone during fruit development has not been reported. However, it is clear that other factors can control fruit dehiscence, because *SHP* proteins and their specific domains are a relatively recent acquisition in core eudicots and in non‐core eudicots, which only have *PaleoAG* genes from dehiscent fruits where valves separate from each other (Pabón‐Mora et al., 2014; Becker, 2020). Such possible factors include the closely related *SEEDSTICK* genes, whose expression defines the dehiscence zone in the basal angiosperm *Aristolochia fimbriata* (Suárez‐Baron et al., 2016). Alternatively, downstream bHLH transcription factors such as *SPT* and *ALC* can be activated independently or autonomously in the absence of *SHP* expression. However, another interesting possibility arises from our data. Because the *P. angustifolia* fruits lack a dehiscence zone and, at late stages, do not open but ripen, *SHP* gene function may not be required in the two developmental stages sampled. However, in other drupaceous fruits, albeit from superior ovaries, like peach, *SHP* genes are expressed, together with *STK* homologs in the endocarp early during fruit transition (Dardick and Callahan, 2014), which would contradict our hypothesis. Nevertheless, no specific roles have been assigned to *SHP* homologs in other fruits outside of core eudicot capsules. Such function may vary among families and in particular in taxa with fruits developing protected by a floral cup like most Rubiaceae.

### *RPL* **homeobox gene homologs in Rubiaceae**

REPLUMLESS, a TALE homeodomain protein, is an important player in the development of the medial domain in the bicarpellate gynoecium derived silique in *Arabidopsis* (Roeder et al., 2003). Since a specific reduction in the replum that is seen in *rpl* mutants is accompanied by ectopic valve margin cells in this medial region, a role in the repression of *SHP* has also been assigned to *RPL* (Roeder et al., 2003, 2005). The gene family to which *RPL* belongs to, has been mostly retained as single copy, with exceptions in Poaceae, Solanaceae, and the Ranunculales (Ortíz‐Ramirez et al., 2018; Zumajo‐Cardona et al., 2018; Appendix S10). Nevertheless, roles assigned to *RPL* homologs include controlling fruit shedding in grasses and specifying the dehiscence zone in poppies (Lin et al., 2007; Arnaud et al., 2011; Zumajo‐Cardona et al., 2018). *RPL* genes also have divergent expression patterns between fleshy and dry fruits in Solanaceae species, because *RPL* is turned off during maturation of fleshy fruits, contrasting with its continuous expression in dry‐fruited taxa (Ortíz‐Ramírez et al., 2018). However, *RPL* homologs in any Solanaceae species have not yet been functionally characterized.

We only found *RPL* homologs in *Condaminea corymbosa* (Ixoroideae) and all species within Rubioideae sampled (Table 1; Appendix S10). Although found as single copy in most species, two copies are present in *Palicourea angustifolia* (Table 1; Appendix S10). The lack of *RPL* orthologs isolated from *Borojoa patinoi, Cephalanthus occidentalis*, either *Coffea* spp., or *Cinchona ledgeriana* suggests species‐specific losses and little or no functional contribution of *RPL* to flower or fruit development in these species.

The expression patterns detected for *RPL* homologs in *C. corymbosa, G. hypocarpium*, and *P. angustifolia* differ. The active expression of *RPL* homologs during carpel and fruit development in *G. hypocarpium* and *P. angustifolia* contrasts with active *RPL* transcripts restricted to carpels of *C. corymbosa* (Figure 6). Based on these data, we suggest that *RPL* is differentially expressed during fleshy versus dry fruit development, but there is not enough data to point to specific roles in the presence of an epicarp. The fact that *RPL* and *SHP* expression does not overlap in species with fleshy epicarps studied suggests that *RPL* genes may be able to repress *SHP* in Rubiaceae to some extent. Nevertheless, spatial resolution in the expression of these genes needs to be performed to support any of these functional hypotheses.

### **The bHLH** *ALC* **/** *SPT* **gene homologs in Rubiaceae**

*ALCATRAZ* and *SPATULA* genes result from a duplication event traced to the core eudicots (Pabón‐Mora et al., 2014; Ortíz‐Ramirez et al., 2018; Appendix S13). Together, *SPT* and *ALC* genes control gynoecium patterning and the development of the separation layer in the *Arabidopsis* fruit (Alvarez and Smyth, 1999; Heisler et al., 2001; Groszmann et al., 2008; Rajani and Sundaresan, 2001). The two genes have opposite expression patterns in dry versus fleshy Solanaceae fruits but act redundantly to repress lignification in time and space independent of fruit type (Ortíz‐ Ramírez et al., 2018, 2019). As expected from the mapping of the core eudicot duplication, most Rubiaceae species have gene representatives in the gene clades *ALC* and *SPT*. However, only *ALC* orthologs were isolated from the *Cephalanthus occidentalis, Borojoa patinoi*, and *Galium hypocarpium* transcriptomes. Similarly, only *SPT* orthologs were found in *Palicourea angustifolia* (Appendix S13).

The expression patterns detected for *ALC*/*SPT* homologs in *C. corymbosa, G. hypocarpium*, and *P. angustifolia* suggest that both *ALC* and *SPT* may play important roles during carpel development in *C. corymbosa*, and only *SPT* orthologs stay active during fruit development in *C. corymbosa* and *P. angustifolia*. However, a putative role in specifying when and how lignification patterns start, like those identified in Solanaceae (Ortíz‐Ramirez et al., 2018, 2019) cannot be readily assigned to *ALC*/*SPT* genes in Rubiaceae. Similar to other gene lineages discussed so far, spatial expression resolution in additional developmental stages as well as more comparative studies in species with different fruit types are required to assess their contribution in Rubiaceae.

### **The bHLH** *HEC* **/** *IND* **gene homologs in Rubiaceae**

Our reconstruction of the *IND*/*HEC* gene phylogeny recovers a first duplication early during angiosperm diversification resulting in the *HEC3*/*IND* and the *HEC1*/*2* clades and a Brassicaceae‐specific duplication resulting in the *HEC3* and *IND* clades (Appendix S16). Same duplication patterns were reported in previous studies (Pabón‐Mora et al., 2014; Pfannebecker et al., 2017; Ortíz‐Ramirez et al., 2018). Because of the duplications mapped, most core eudicots have *HEC1*/*2*/*3* homologs but lack *IND* orthologs. In *Arabidopsis, INDEHISCENT* (*IND*) controls lignification in the dehiscence zone and, in turn, the separation between the valve margins and the lignified layers (Liljegren et al., 2004). On the other hand, *HECATE1*/*2*/*3* genes control style, stigma, and transmitting tract development (Gremski et al., 2007; Schuster et al., 2015). Considering that these roles are crucial for plant reproduction, it is interesting that other *HEC* gene expression in Solanaceae is largely absent from gynoecium in different species, except perhaps for *HEC3* homologs, which remain broadly expressed during carpel and fruit development (Ortíz‐Ramirez et al., 2018).

In Rubiaceae, we were able to isolate one *HEC* ortholog of each clade only in *Coffea arabica* and *Galium hypocarpium*. All other species conserved only one of the three copies, in particular, the ortholog *HEC3*. No *HEC* homologs were isolated in *Condaminea corymbosa*. Expression of *HEC* homologs in *Galium hypocarpium* and *Palicourea angustifolia* was found in carpels and fruits (Figure 6). These results suggest some contribution of *HEC* homologs in gynoecium patterning in Rubiaceae, more similar to the data gathered in Brassicaceae, than Solanaceae, and some roles in fruit development. The fact that massive stigmas, thick styles, and very conspicuous transmitting tracts are present regularly in Rubiaceae merits an assessment of the contribution of *HEC* genes to the formation of these structures in the family.

### Shifts in genetic control of carpel/fruit development in Rubiaceae in relation to variation in fruit type

Our data provide the first compilation of carpel/fruit genes in the Rubiaceae and point to specific transcription factors active during carpel versus fruit development in three selected species. Most canonical genes described in *Arabidopsis* have homologs in Rubiaceae. The genetic complement in all the gene lineages resembles that reported for other core eudicots (Pabón‐Mora et al., 2014; Pfannebecker et al., 2017), perhaps with a notorious reduction in *RPL* genes. Unlike the Brassicaceae and the Solanaceae, the Rubiaceae lack any traces of WGD events increasing gene copy number in the family (Denoeud et al., 2014). Our findings support the lack of WGD, as none of the gene families explored, seems to have undergone Rubiaceae specific duplications (Table 1; Appendices S4, S7, S10, S13, S16). Thus, the Rubiaceae provide an excellent system in which to explore gene expression and function underlying the diversity of fruit types in angiosperms. In particular, those derived from inferior ovaries with the recruitment of floral tissue during development. The expression analyses suggest that different species may be using the genes in the network differently. For example, while most genes isolated here are active during carpel and fruit transition in *Galium hypocarpium*, fewer genes are transcribed in the same stages in *Condaminea corymbosa*. In the latter, only *SHP, RPL* and the *ALC*/*SPT* genes play roles in carpel development, and only *SHP* and *SPT* homologs are active during the transition to fruit. This network is notoriously reduced compared to that of *Arabidopsis*.

Our data also points to abundant changes in the functional evolution of the gene homologs among Rubiaceae species. In *Palicourea angustifolia, FUL, AG, RPL*, and *HEC2* homologs seem to play roles in both carpel and fruit development, and only *SPT* and *HEC1* are turned on exclusively in early stages of fruit development. Although comparative expression studies by RT‐PCR underestimate the full diversity in spatiotemporal expression and only help to frame incipient functional hypotheses, they provide a hint of the extensive functional diversity of these transcription factors in the patterning of carpels and fruits. However, it is also important to consider that the reduced number of genes active in fruit development may be a consequence of histological specialization coming from the epicarp (floral cup derived) and not from the pericarp (fruit wall derived). In consequence, other candidate genes, besides the *Arabidopsis* canonical fruit genes, may be playing important roles. Among those that stand out are the *SEPALLATA* genes, identified in strawberry and apple development (Seymour et al., 2011; Ireland et al., 2013). Future studies will have to target a more comprehensive set of transcription factors different from the canonical and most intensively studied transcription factors from *Arabidopsis* and other emerging model core eudicots.

## CONCLUSIONS

Fruit diversity in Rubiaceae is the result of (1) the combination of ovary‐derived and floral cup tissues and (2) the different and independent cellular process that can occur in the two tissues during fruit maturation. Strictly speaking, the fruits of *C. corymbosa* and *P. angustifolia* correspond to lignified pericarps with fleshy epicarps, while the fruits of *G. hypocarpium* and *M. citrifolia* correspond to fleshy pericarps covered by fleshy epicarps. The present study is the first systematic isolation of fruit development genes in Rubiaceae from seven reference transcriptomes. Our results show that, unlike other asterids, the Rubiaceae lack traces of whole‐genome duplication events at the family level. Although both species‐specific duplications and losses are here reported, the latter can only be confirmed in the future with full‐genome sequencing. Our expression analyses suggest that different species may be using a slightly different genetic network, where *FUL* may retain important roles in fruit wall development in some species, while *SHP, ALC, RPL*, and *HEC* seem to have more flexible roles in fruit development in the Rubiaceae. Additionally while in general *FUL, AG, RPL*, and *HEC2* are active in carpels and fruits, *SPT* and *HEC1* homologs are exclusive to early stages of fruit development. Furthermore, we detect expression patterns that suggest a different genetic regulatory network in the histogenesis of Rubiaceae fruits compared to Brassicaceae. For instance, (1) *FUL* genes are inactive in early and late stages of fruit development in *Condaminea corymbosa*; (2) *SHP* orthologs, in the absence of *AG* homologues, may be compensating for important roles in the identity of stamens and carpels in the same species; and (3) the bHLH homologs *SPT* and *ALC* are expressed in the absence of *SHP* in *Palicourea angustifolia*. All of our hypotheses based on expression patterns, however, must be tested with more detailed analyses of spatiotemporal expression and function. This study constitutes the first comprehensive investigation of the morphoanatomy and genetics of fruit development in Rubiaceae, a family with high economic and ethnobotanical value.

## AUTHOR CONTRIBUTIONS

H.S.D., A.I.U., and N.P.‐M. designed the research project and framed the experimental design; H.S.D. and N.P.‐M. did fieldwork, performed all the experiments, organized the data and wrote the first draft of the manuscript; J.F.A. assembled all transcriptomes and performed BLAST searches; A.I.U., C.F., and N.P.‐M. secured funding for the research. All authors analyzed the data and revised and approved the content of the manuscript.

## Supporting information


**Appendix S1**. Transcriptomic statistics for all sevenspecies of Rubiaceae selected.

**Appendix S2**. List of gene names, corresponding contigs, and databases sampled for all sequences identified in this study and those used in all phylogenetic analyses.

**Appendix S3**. Primers used to amplify all gene copies from fruit patterning genes in *Condaminea corymbosa, Galium hypocarpium*, and *Palicourea angustifolia*.

**Appendix S4**. Maximum likelihood analysis of *AP1/FUL‐like* genes. Big yellow stars indicate large‐scale duplication events in core and basal eudicots. Small red stars indicate local duplication events at the order/family level. Small gray stars point to species‐specific duplications. Purple branches are used to represent all the genes belonging to Rubiaceae species and blue branches correspond to Brassicaceae homologs. Ultra‐Fast Bootstrap values are shown at nodes.

**Appendix S5**. Comparative analysis of conserved motifs in 39 selected euFULI and euFULII proteins. All the conserved motifs were identified using MEME suite. Colored boxes indicate motifs 1 to 12. Protein names and combined probability values are shown on the left.

**Appendix S6**. Sequences of the conserved motifs detected on the FUL‐like proteins in angiosperms. The MADS‐box protein motifs MADS‐box, I‐region, K‐box, and C‐region are underlined. Conserved sites within are dashed.

**Appendix S7**. Maximum likelihood analysis of *AG*/*SHP‐like* genes. Duplication events are indicated by the starts. Star and branch colors follow the same conventions indicated in Figure 2. Ultra‐Fast Bootstrap values are shown at the nodes.

**Appendix S8**. Comparative analysis of conserved motifs in 32 selected AG/SHP proteins. All the conserved motifs were identified using MEME suite. Colored boxes indicate motifs 1 to 12. Protein names and combined probability values are shown on the left.

**Appendix S9**. Sequences of the conserved motifs detected on the *AG*/*SHP* homologues in angiosperms. The MADS‐box protein motifs MADS‐box, I‐region, K‐box, and C‐region are underlined. AG motifs I and II in the C‐terminal domain are highlighted.

**Appendix S10**. Maximum likelihood analysis of *RPL* genes. Duplication events are indicated by the starts. Star and branch colors follow the same conventions indicated in Figure 2. Ultra‐Fast Bootstrap values are shown at nodes.

**Appendix S11**. Comparative analysis of conserved motifs from 10 selected RPL proteins. All the conserved motifs were identified using MEME suite. Colored boxes indicate motifs 1 to 12. Protein names and combined probability values are shown on the left.

**Appendix S12**. Sequences of the conserved motifs detected on the RPL homologs in angiosperms. Two main domains are shown: the BELL domain and the complete sequence on the homeodomain. The dashed rectangles indicate the consensus from the typical protein domains for the SKY, BELL, the three amino acid loop helixes (HD) and the ZIBEL box motifs.

**Appendix S13**. Maximum likelihood analysis of *ALC*/*SPT* genes. The stars indicate duplication events. Star and branch colors follow the same conventions indicated in Figure 2. Ultra‐Fast Bootstrap values are shown at nodes.

**Appendix S14**. Comparative analysis of conserved motifs in 21 selected ALC/SPT proteins. All the conserved motifs were identified using MEME suite. Colored boxes indicate motifs 1 to 12. Protein names and combined probability values are shown on the left.

**Appendix S15**. Sequences of the conserved motifs detected on the ALC/SPT homologs in angiosperms. The bHLH domain follows Groszmann et al. ( 2011). Rubiaceae specific motifs are highlighted. Black lines point to highly conserved sites in these consensus sequences.

**Appendix S16**. Maximum likelihood analysis of *HEC*/*IND* genes. Duplication events are indicated by the starts. Star and branch colors follow the same conventions indicated in Figure 2. Ultra‐Fast Bootstrap values are shown at nodes.

**Appendix S17**. Comparative analysis of conserved motifs in 24 selected HEC/IND proteins. All the conserved motifs were identified using MEME suite. Colored boxes indicate motifs 1 to 12. Protein names and combined probability values are shown on the left.

**Appendix S18**. Sequences of the conserved motifs detected on the HEC/IND homologues in angiosperms. Black line on the left in motif 1 of the bHLH domain shows the HEC domain identified by Kay et al. ( 2013), the conserved regions of bHLH, and its beta strand tale. Dashed rectangles indicate de NLS region inside of the Helix 1 in the bHLH domain.

**Appendix S19**. Positive controls for PCR amplifications from mixed cDNA of all genes that were not detected at S0 or S1.

## Data Availability

All sequences isolated in this work are available under GenBank numbers MW356675‐MW356760.
