# Supplementary material for: Comparative anatomy and genetic bases of fruit development in selected Rubiaceae (Gentianales)
Source: Am J Bot. 2021 Oct 26;108(10):1838–60. doi: 10.1002/ajb2.1785 (PMC9298371; doi:10.1002/ajb2.1785)
Supplement: Supplementary file 1 — Appendix S1. Transcriptomic statistics for all sevenspecies of Rubiaceae selected. [file AJB2-108-1838-s006.docx]

**Salazar-Duque et al.—American Journal of Botany 2021**

**Appendix S1.** Transcriptomic metrics of the selected Rubiaceae species.

| ***Borojoa patinoi*** | Trinity_clean_Bopa.Trinity. fasta_STATS  Total length of sequence: 52,310,506 bp  Total number of sequences: 56,872  Average contig length: 919 bp  Largest contig: 8186 bp  Shortest contig: 201 bp  N25 stats: 25% of total sequence length is contained in the 4927 sequences ≥ 2073 bp  N50 stats: 50% of total sequence length is contained in the 12,470 sequences ≥ 1457 bp  N75 stats: 75% of total sequence length is contained in the 23,975 sequences ≥ 847 bp  Total GC count: 22,424,549 bp  GC %: 42.87 |
| --- | --- |
| ***Cephalanthus occidentalis*** | Trinity_clean_Cepha.Trinity. fasta_STATS  Total length of sequence: 41,431,312 bp  Total number of sequences: 51,880  Average contig length: 798 bp  Largest contig: 5307 bp  Shortest contig: 201 bp  N25 stats: 25% of total sequence length is contained in the 6179 sequences ≥ 1398 bp  N50 stats: 50% of total sequence length is contained in the 14,578 sequences ≥ 1089 bp  N75 stats: 75% of total sequence length is contained in the 25,904 sequences ≥ 741 bp  Total GC count: 17,210,417 bp  GC %: 41.54 |
| ***Coffea arabica*** | Trinity_clean_Coar.Trinity. fasta_STATS  Total length of sequence: 124,406,074 bp  Total number of sequences: 103,789  Average contig length: 1198 bp  Largest contig: 8143 bp  Shortest contig: 201 bp  N25 stats: 25% of total sequence length is contained in the 9884 sequences ≥ 2481 bp  N50 stats: 50% of total sequence length is contained in the 24,746 sequences ≥ 1784 bp  N75 stats: 75% of total sequence length is contained in the 46,084 sequences ≥ 1155 bp  Total GC count: 52,281,377 bp  GC %: 42.02 |
| ***Condaminea corymbosa*** | Trinity_clean_Cory. Trinity. Fasta STATS  Total length of sequence: 58,565,181 bp  Total number of sequences: 63,105  Average contig length: 928 bp  Largest contig: 9342 bp  Shortest contig: 201 bp  N25 stats: 25% of total sequence length is contained in the 5521 sequences ≥ 2072 bp  N50 stats: 50% of total sequence length is contained in the 14,059 sequences ≥ 1433 bp  N75 stats: 75% of total sequence length is contained in the 27,219 sequences ≥ 826 bp  Total GC count: 24,846,192 bp  GC %: 42.42 |
| ***Galium hypocarpium*** | Trinity_clean_Gahy. Trinity. fasta_STATS  Total length of sequence: 117,610,932 bp  Total number of sequences: 127,371  Average contig length: 923 bp  Largest contig: 15,570 bp  Shortest contig: 201 bp  N25 stats: 25% of total sequence length is contained in the 8884 sequences ≥ 2371 bp  N50 stats: 50% of total sequence length is contained in the 24,594 sequences ≥ 1501 bp  N75 stats:  75% of total sequence length is contained in the 51,234 sequences ≥ 777 bp  Total GC count: 51,907,773 bp  GC %: 44.14 |
| ***Morinda citrifolia*** | Trinity clean Moci. fasta_STATS  Total length of sequence: 161,582,814 bp  Total number of sequences: 130,936  Average contig length: 1234 bp  Largest contig: 15,484 bp  Shortest contig: 201 bp  N25 stats: 25% of total sequence length is contained in the 10,619 sequences ≥ 2845 bp  N50 stats: 50% of total sequence length is contained in the 27,995 sequences ≥ 1928 bp  N75 stats: 75% of total sequence length is contained in the 54,613 sequences ≥ 1159 bp  Total GC count: 65,622,136 bp  GC %: 40.61 |
| ***Palicourea angustifolia*** | Trinity_ clean Pali. fasta_STATS  Total length of sequence: 174,935,973 bp  Total number of sequences: 154,399  Average contig length: 1133 bp  Largest contig: 12,282 bp  Shortest contig: 201 bp  N25 stats: 25% of total sequence length is contained in the 12,274 sequences ≥ 2678 bp  N50 stats: 50% of total sequence length is contained in the 32,497 sequences ≥ 1786 bp  N75 stats: 75% of total sequence length is contained in the 63,783 sequences ≥ 1059 bp  Total GC count: 73,216,981 bp  GC %: 41.85 |
