# Supplementary material for: Comparative anatomy and genetic bases of fruit development in selected Rubiaceae (Gentianales)
Source: Am J Bot. 2021 Oct 26;108(10):1838–60. doi: 10.1002/ajb2.1785 (PMC9298371; doi:10.1002/ajb2.1785)
Supplement: Supplementary file 3 — Appendix S3. Primers used to amplify all gene copies from fruit patterning genes in Condaminea corymbosa, Galium hypocarpium, and Palicourea angustifolia. [file AJB2-108-1838-s004.docx]

**Salazar-Duque et al.—American Journal of Botany 2021**

**Appendix S3.** Primers used for gene amplification by RT-PCR in *Condaminea corymbosa* (Coco), *Galium hypocarpium* (Gahy), and *Palicourea angustifolia* (Paan).

| **Primer ID** | **Sequence** |
| --- | --- |
| ***Condaminea corymbosa*** | |
| CocoSHP1-Rev | GGTCCTGGCGAGAGTAATGCT |
| CocoSHP2-Rev | TGGTCCTGATAATGTTGATCA |
| CocoSHP1-fwd | ATGGCTTGTCCTACTCAAGAT |
| CocoSHP2-fwd | ATGGCTTGTCCTAATCAACAT |
| CocoRPL-Fwd | CAGATAAAGTAATGTTGGCTAA |
| CocoRPL-Rev | ATCATGCAAGAGTTGCCCTCC |
| CocoALC-Fwd | AATGGACTCGGTTTTCATCCA |
| CocoALC-Rev | CTATGAAGACACACCTGATGA |
| CocoSPT-Fwd | ATGTGTCTGCCTGGAGTGTTG |
| CocoSPT-Rev | CCCATCCACTTGTGACAAAAG |
| ***Galium hypocarpium*** | |
| GahyAG1_2-Fwd | TGAAAGCGAGCATGAGCGTTCC |
| GahyAG1-Rev | GGCATCAGATTCATGTGCTGC |
| GahyAG2-Rev | TGCTCGAAGATATTGATTCAT |
| GahyRPL-Fwd | CGAGCGTGCTGTCACTGTTCT |
| GahyRPL-Rev | ATCGCCTTGTGGAATTCATCG |
| GahyALC-Fwd | ATGATTAATTCCTCCGCCGCT |
| GahyALC-Rev | ATCCGAGTCCATTTCTCATCG |
| GahyHEC1-Fwd | GTATCGATAAAGCCGCCGCGG |
| GahyHEC1-Rev | TCACTGACGCCGACGCCGACG |
| GahyHEC2-Fwd | ATGATGCTGATGATGCAGATG |
| GahyHEC2-Rev | GATGTGAATCGGTTGCATTGC |
| GahyHEC3-Fwd | ACGAATTACAGCCCATGGGAT |
| GahyHEC3-Rev | GACGACATGTTGGCCACTGCA |
| GahyAP1_1_2-Fwd | GAGCATCCTGGAGCGCTATGA |
| GahyAP1_1-Rev | ACAGTGTTAGAAAACCTACTC |
| GahyAP1_2-Rev | GTAAGATCTAGTAGTTCATTC |
| GahyFUL(1/5)-Fwd | AGGTACTCCCATGCAGAAATG |
| GahyFUL1-Rev | CAGCAGGCTTGATAATCCATA |
| GahyFUL2-Rev | GTTGCGCTATTAGTATTAGTA |
| GahyFUL3-Rev | CATCCAGGAAGGCATCGTTGC |
| GahyFUL5-Rev | CGTTAAGAAGTTCATACCTGT |
| ***Palicourea angustifolia*** | |
| PaanAP1-4Fwd | ATGGAGAGTATCCTAGAACGG |
| PaanAP1_1-Rev | TCAGGCAGCAAAGCATCCCAG |
| PaanAP1_2-Rev | CGTCAGGCAGCAAAGCATCCC |
| PaanAP1_3-Rev | CATTATATATAGTTCTGAATA |
| PaanAP1_4-Rev | TAGGCAAGGAGGGATAGTCTG |
| PaanFUL1-fwd | TCGTGAACACCATGAGATGGA |
| PaanFUL2-fwd | TGTAGAACTTCAATCCTAGCC |
| PaanFUL3-fwd | GAAGAACTGGAGGGCCTTAGT |
| PaanFUL3-Rev | TCATATGGCGAAGCATCCATG |
| PaanAG1-fwd | TCTCCTGAAGATCTATGTGTG |
| PaanAG2&3-fwd | ATGGCGTACCAAAGTGATCAT |
| PaanSHP1&2-fwd | AATCAAGTAGAATTTGAGTCC |
| PaanSHP3-fwd | TCAAAACTGTACAGTTGTTGC |
| PaanAG1-Rev | TCAGACTAATTGAAGAGAGGG |
| PaanAG2-Rev | TAGGATGGAATCAACAAATAACTA |
| PaanAG3-Rev | CATCATATGGCTGAGGTGACA |
| PaanSHP1-Rev | ATGGAGAGTATCCTAGAACGG |
| PaanSHP2-Rev | TCAGGCAGCAAAGCATCCCAG |
| PaanSHP3-Rev | CGTCAGGCAGCAAAGCATCCC |
| PaanRPL1-Fwd | GCTGGAAGTGGAACTGATGAT |
| PaanRPL2-Fwd | CAAAGAGAGGAGCAAACTGGC |
| PaanRPL1-Rev | TGGCGTAATGACTGCGTATCC |
| PaanRPL2-Rev | CATTAGCATCCAGGCCGAACC |
| PaanSPT1&2-Fwd | AGACTGACAAGGCGTCCATGC |
| PaanSPT1-Rev | ACATGCTTCGAGATTGTGTTC |
| PaanSPT2-Rev | CTACTTTGGTGGATTATACGC |
| PaanHEC1-Fwd | ATGGAAACACTGATGCACCTG |
| PaanHEC2-Fwd | TGCCGCAATGCAACCGGTAGA |
| PaanHEC1-Rev | TGCATCGAGCCCACCACGTGA |
| PaanHEC2-Rev | ATGGCCAAGTTGGTGGAGGAG |
|  |  |
| ***ACTIN* for all three species** | |
| ACTIN Rubi-Fwd | GGATTTCCAGGGCTGAATATGA |
| ACTIN Rubi-Rev | CGATACGATAGTAGCAGCTTAAAAGC |
