# Supplementary figures and images for: Comparative anatomy and genetic bases of fruit development in selected Rubiaceae (Gentianales)
Source: Am J Bot. 2021 Oct 26;108(10):1838–60. doi: 10.1002/ajb2.1785 (PMC9298371; doi:10.1002/ajb2.1785)

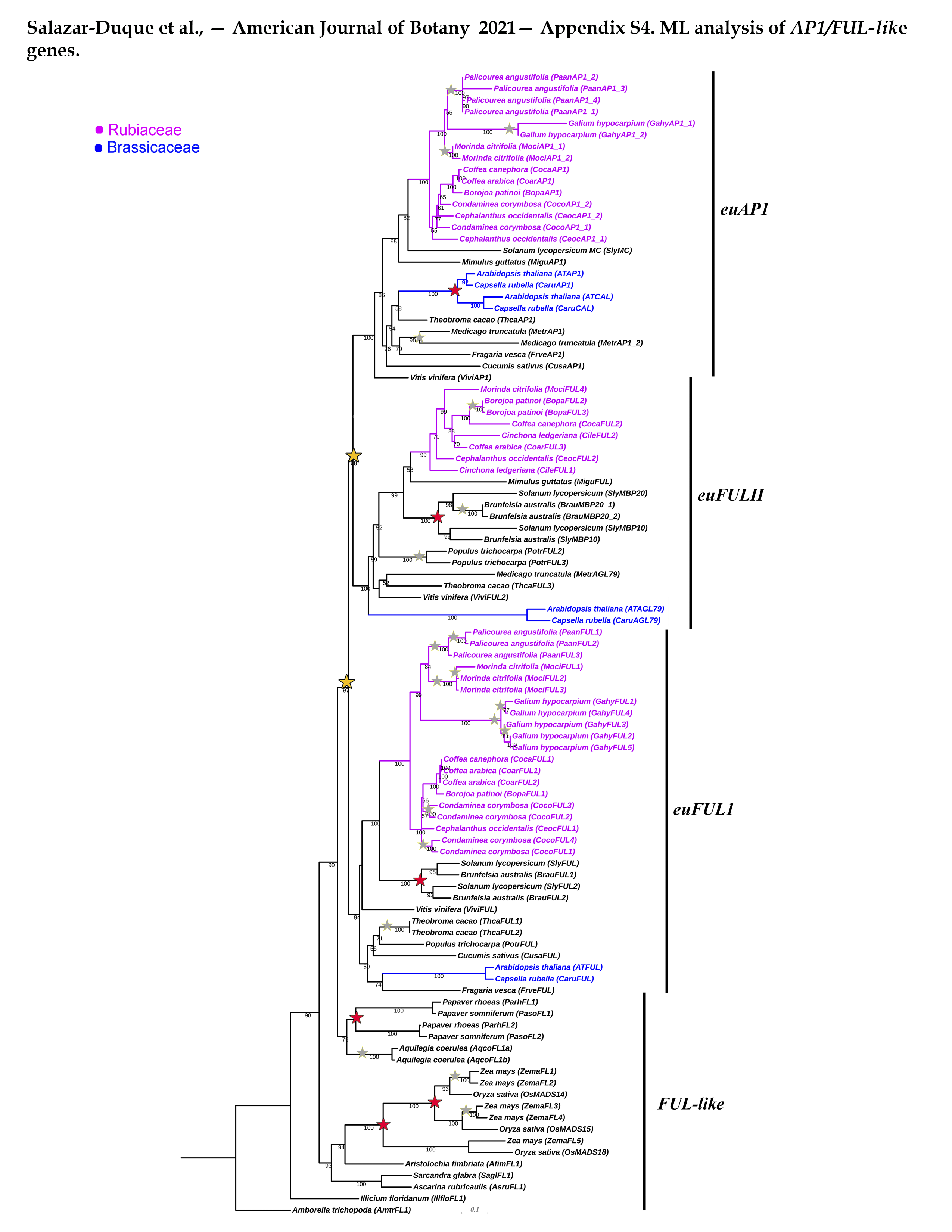

Supplement: Supplementary file 4 — Appendix S4. Maximum likelihood analysis of AP1/FUL‐like genes. Big yellow stars indicate large‐scale duplication events in core and basal eudicots. Small red stars indicate local duplication events at the order/family level. Small gray stars point to species‐specific duplications. Purple branches are used to represent all the genes belonging to Rubiaceae species and blue branches correspond to Brassicaceae homologs. Ultra‐Fast Bootstrap values are shown at nodes. [file AJB2-108-1838-s014.tif]

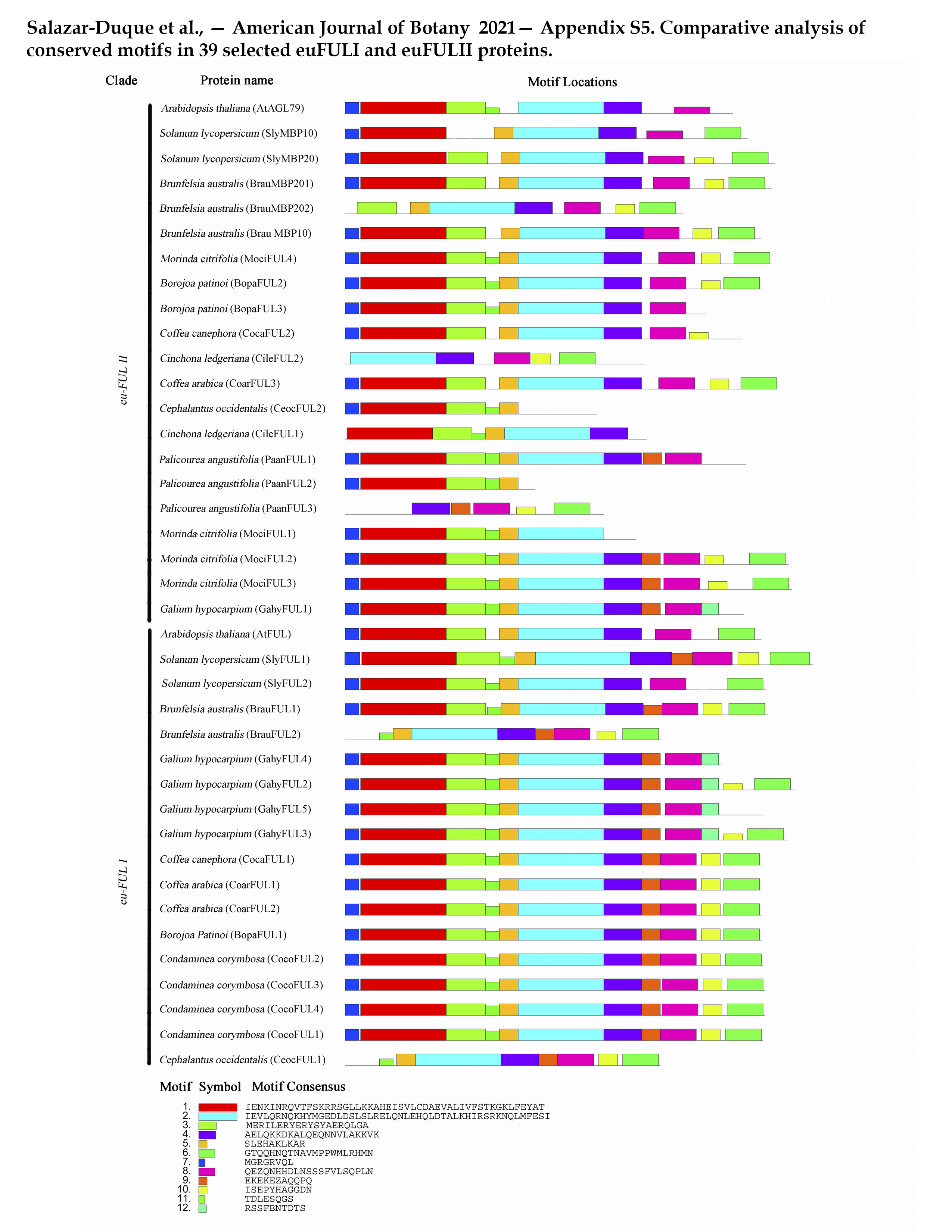

Supplement: Supplementary file 5 — Appendix S5. Comparative analysis of conserved motifs in 39 selected euFULI and euFULII proteins. All the conserved motifs were identified using MEME suite. Colored boxes indicate motifs 1 to 12. Protein names and combined probability values are shown on the left. [file AJB2-108-1838-s007.tif]

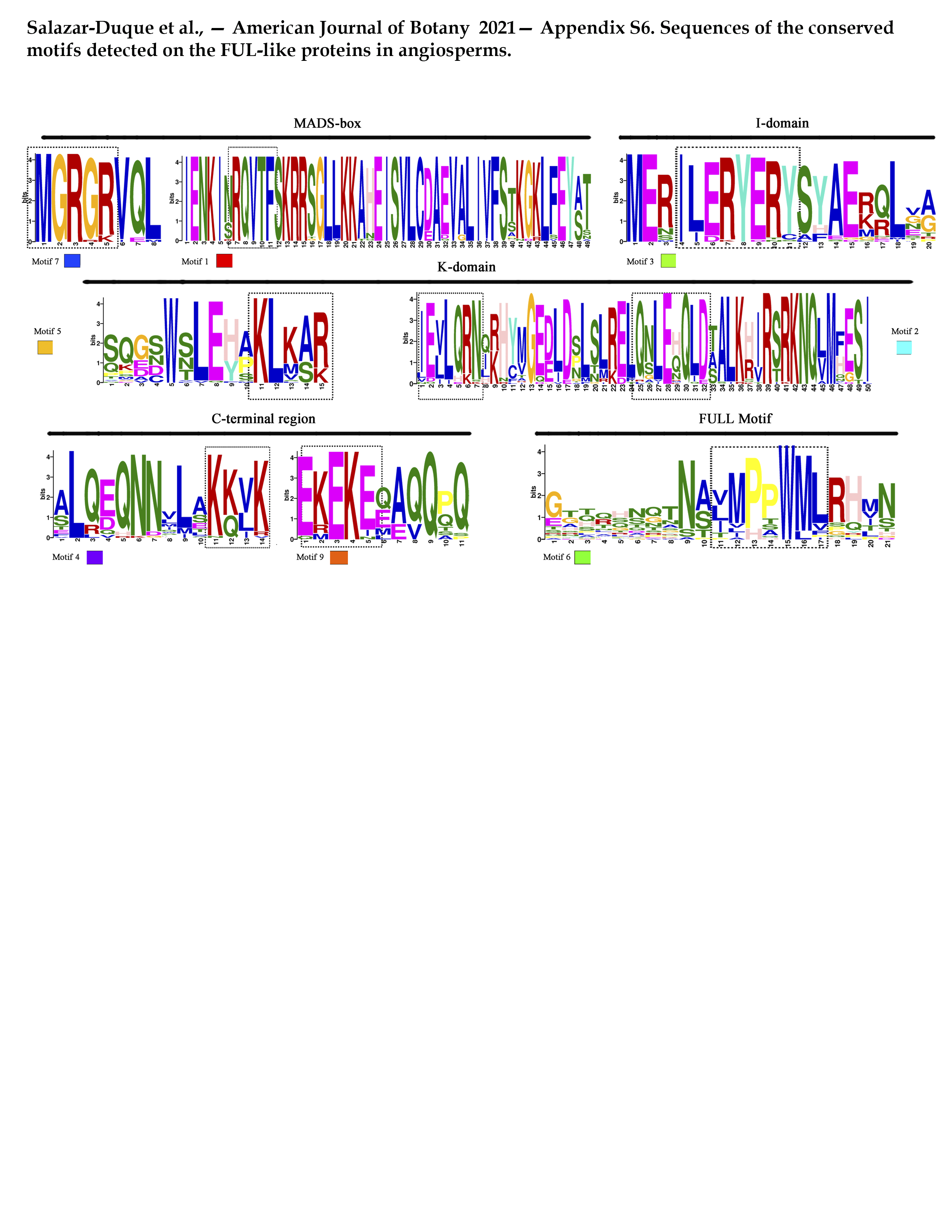

Supplement: Supplementary file 6 — Appendix S6. Sequences of the conserved motifs detected on the FUL‐like proteins in angiosperms. The MADS‐box protein motifs MADS‐box, I‐region, K‐box, and C‐region are underlined. Conserved sites within are dashed. [file AJB2-108-1838-s005.tif]

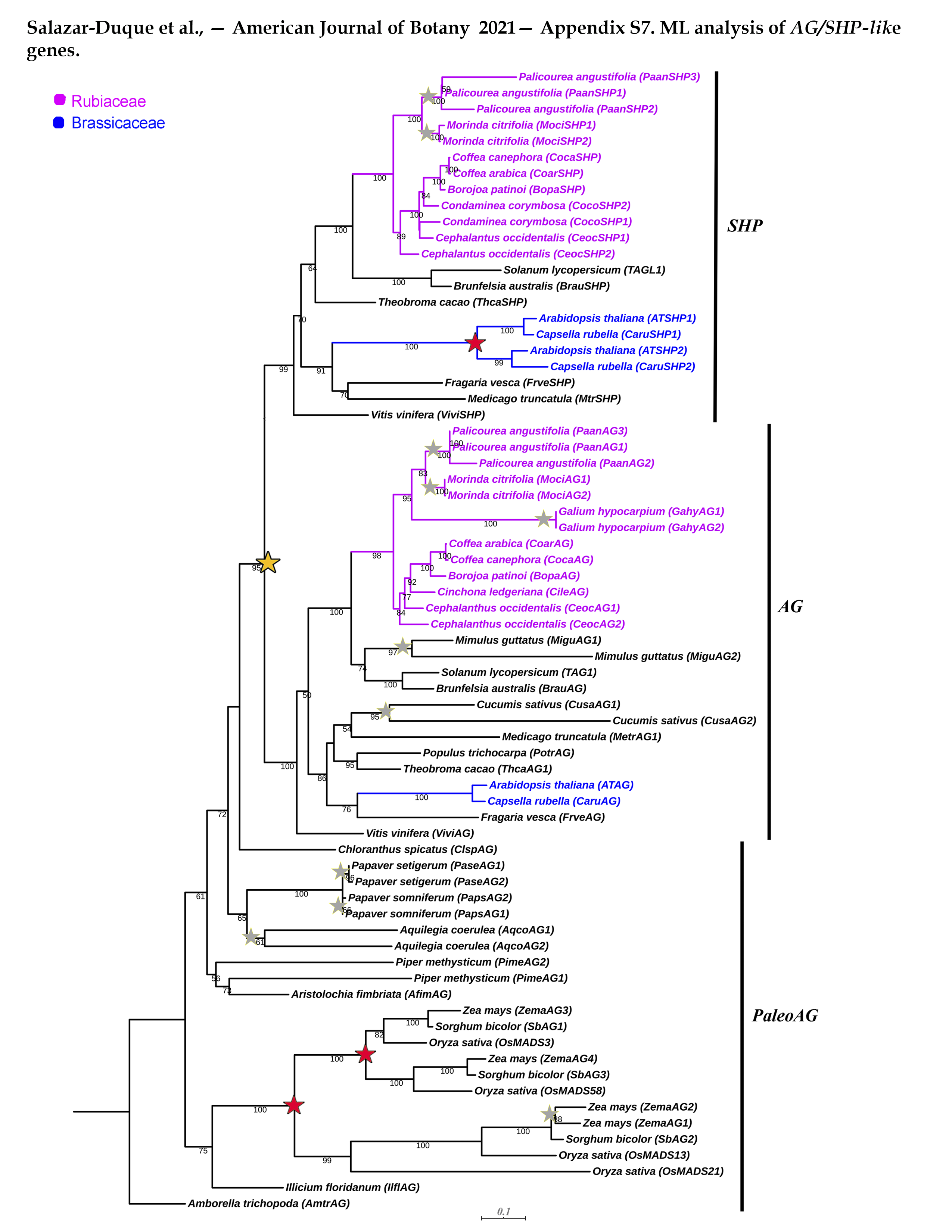

Supplement: Supplementary file 7 — Appendix S7. Maximum likelihood analysis of AG/SHP‐like genes. Duplication events are indicated by the starts. Star and branch colors follow the same conventions indicated in Figure 2. Ultra‐Fast Bootstrap values are shown at the nodes. [file AJB2-108-1838-s017.tif]

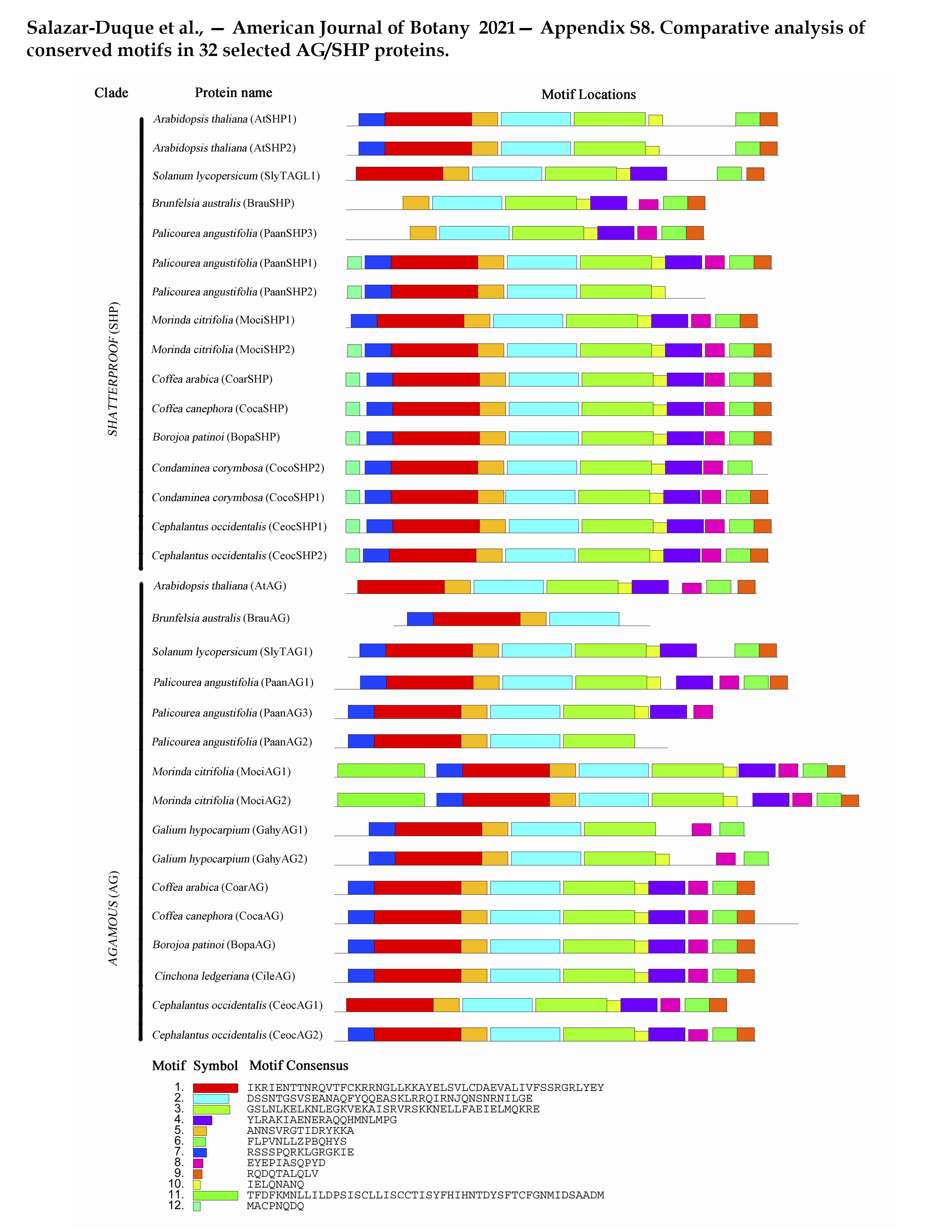

Supplement: Supplementary file 8 — Appendix S8. Comparative analysis of conserved motifs in 32 selected AG/SHP proteins. All the conserved motifs were identified using MEME suite. Colored boxes indicate motifs 1 to 12. Protein names and combined probability values are shown on the left. [file AJB2-108-1838-s011.tif]

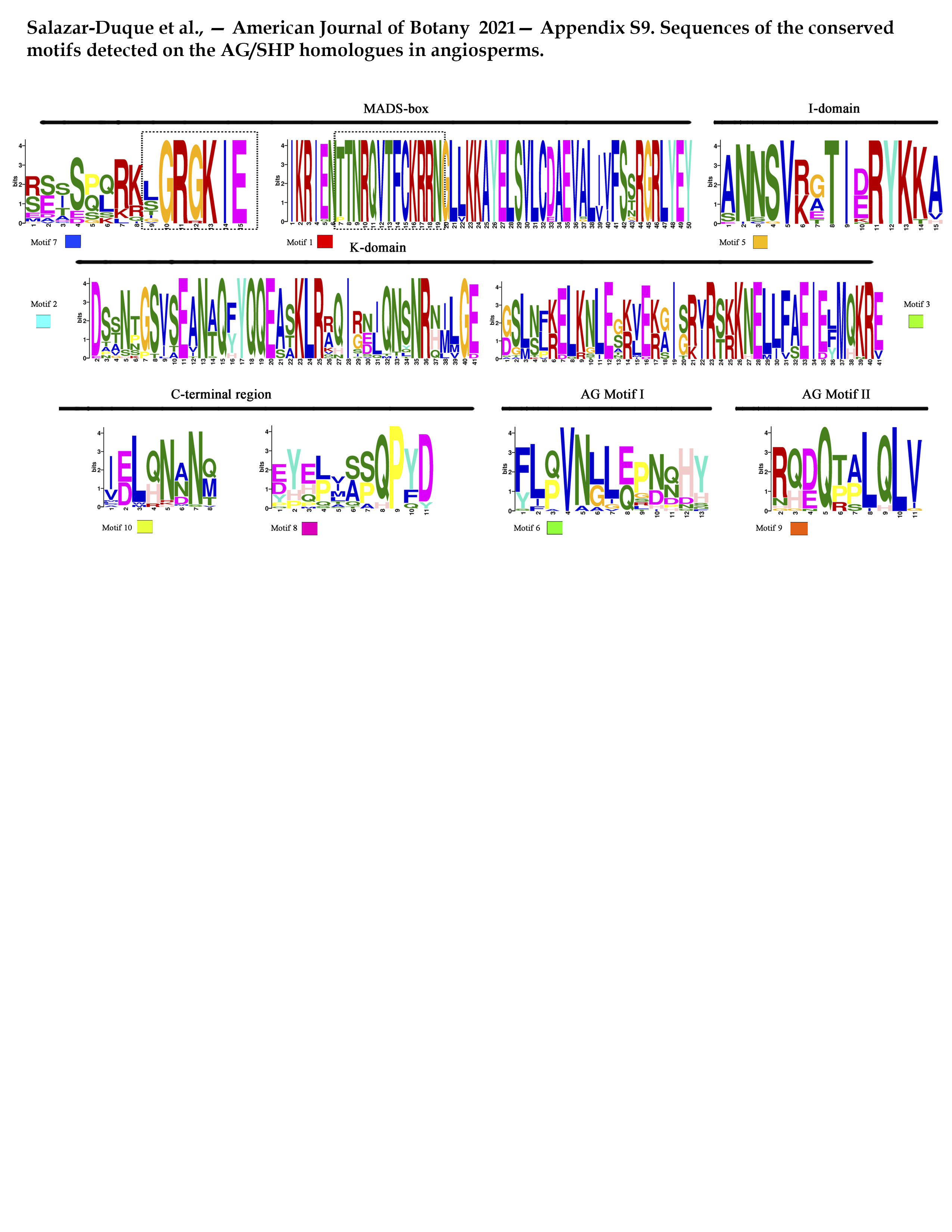

Supplement: Supplementary file 9 — Appendix S9. Sequences of the conserved motifs detected on the AG/SHP homologues in angiosperms. The MADS‐box protein motifs MADS‐box, I‐region, K‐box, and C‐region are underlined. AG motifs I and II in the C‐terminal domain are highlighted. [file AJB2-108-1838-s001.tif]

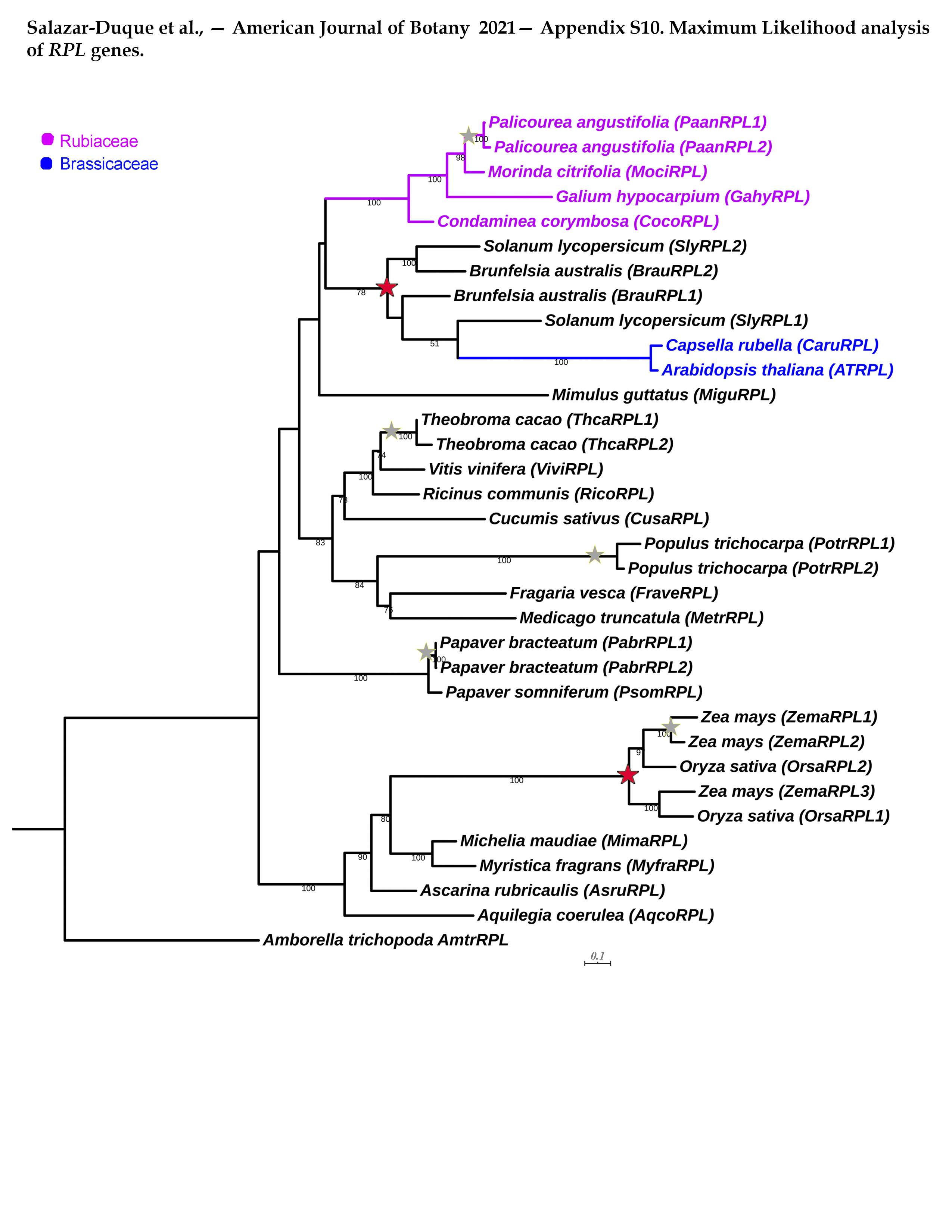

Supplement: Supplementary file 10 — Appendix S10. Maximum likelihood analysis of RPL genes. Duplication events are indicated by the starts. Star and branch colors follow the same conventions indicated in Figure 2. Ultra‐Fast Bootstrap values are shown at nodes. [file AJB2-108-1838-s012.tif]

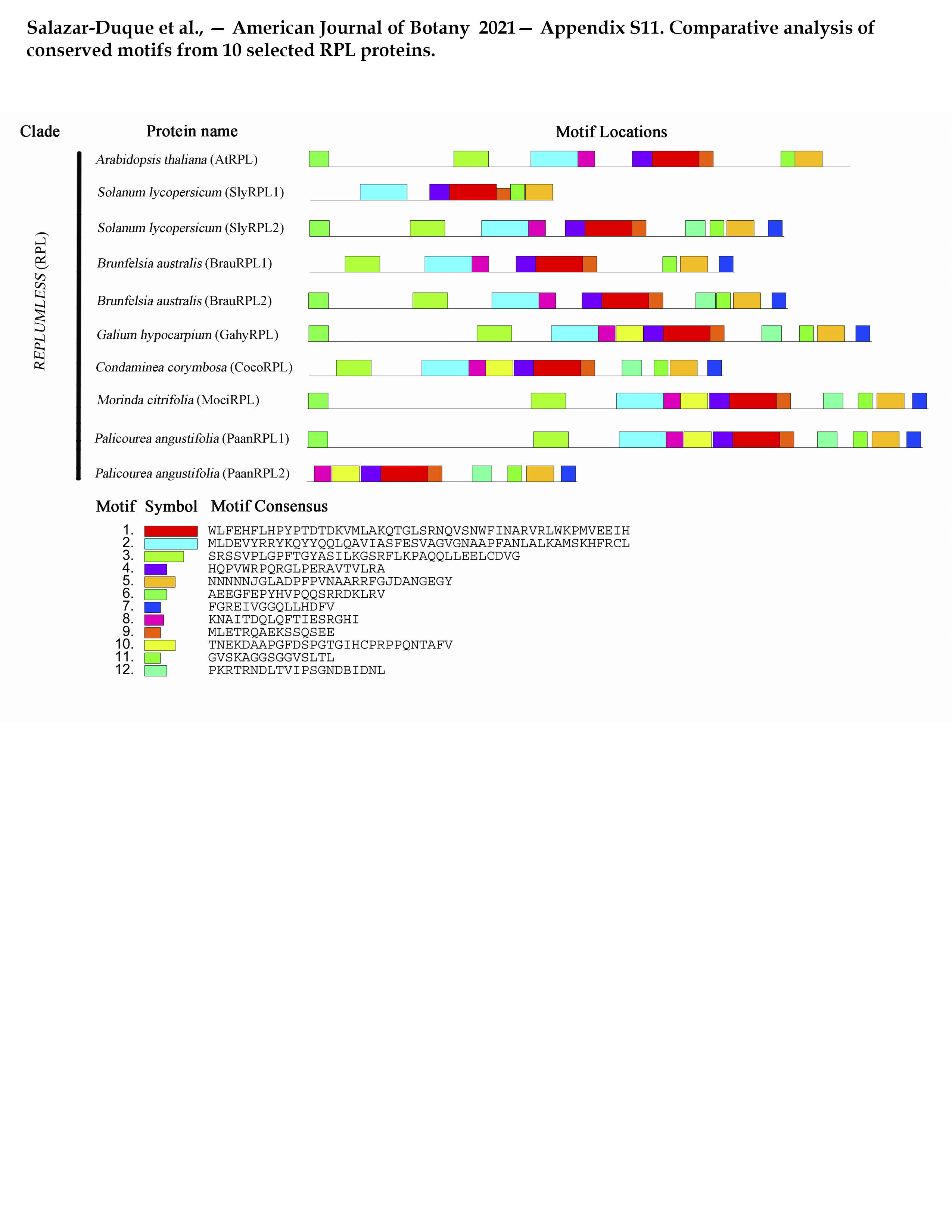

Supplement: Supplementary file 11 — Appendix S11. Comparative analysis of conserved motifs from 10 selected RPL proteins. All the conserved motifs were identified using MEME suite. Colored boxes indicate motifs 1 to 12. Protein names and combined probability values are shown on the left. [file AJB2-108-1838-s008.tif]

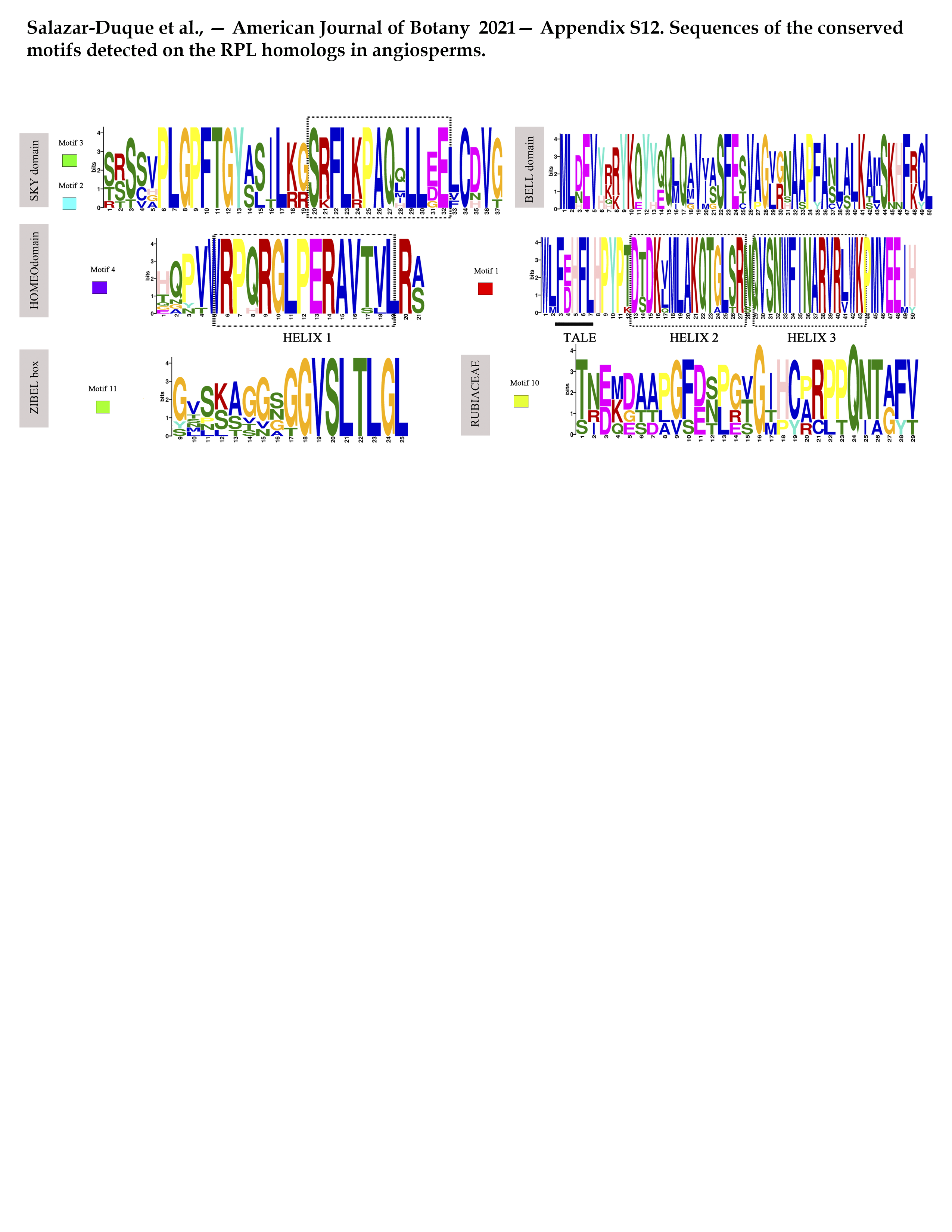

Supplement: Supplementary file 12 — Appendix S12. Sequences of the conserved motifs detected on the RPL homologs in angiosperms. Two main domains are shown: the BELL domain and the complete sequence on the homeodomain. The dashed rectangles indicate the consensus from the typical protein domains for the SKY, BELL, the three amino acid loop helixes (HD) and the ZIBEL box motifs. [file AJB2-108-1838-s013.tif]

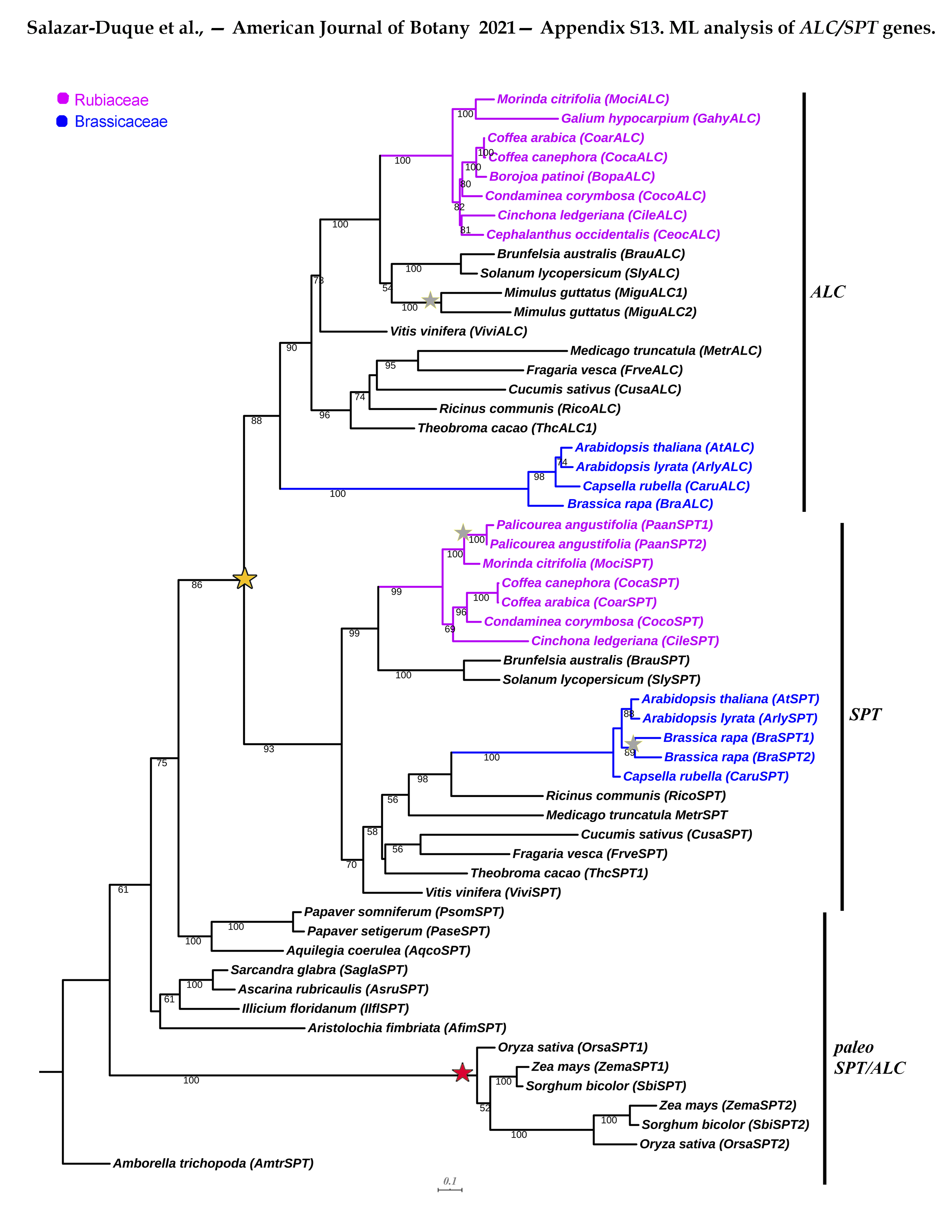

Supplement: Supplementary file 13 — Appendix S13. Maximum likelihood analysis of ALC/SPT genes. The stars indicate duplication events. Star and branch colors follow the same conventions indicated in Figure 2. Ultra‐Fast Bootstrap values are shown at nodes. [file AJB2-108-1838-s018.tif]

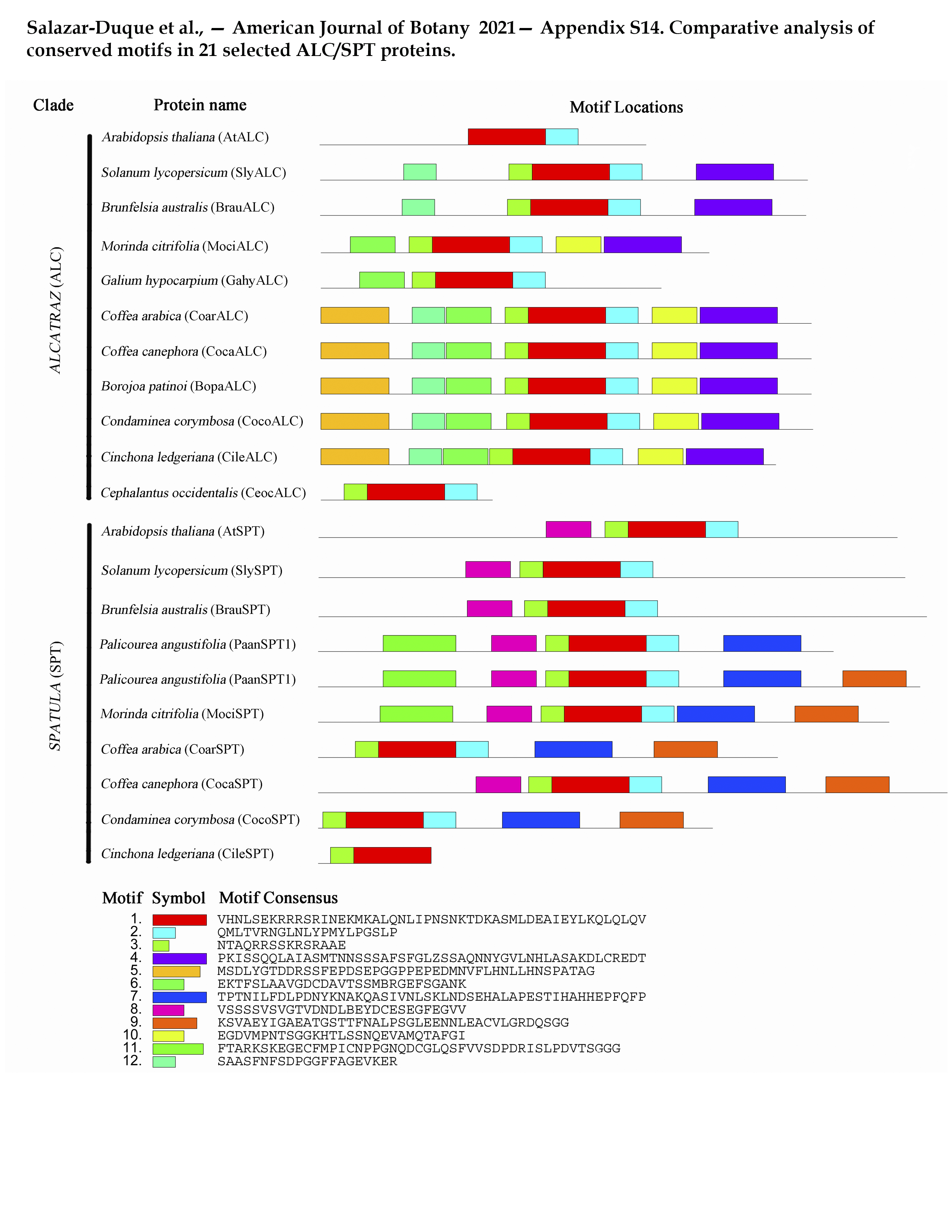

Supplement: Supplementary file 14 — Appendix S14. Comparative analysis of conserved motifs in 21 selected ALC/SPT proteins. All the conserved motifs were identified using MEME suite. Colored boxes indicate motifs 1 to 12. Protein names and combined probability values are shown on the left. [file AJB2-108-1838-s002.tif]

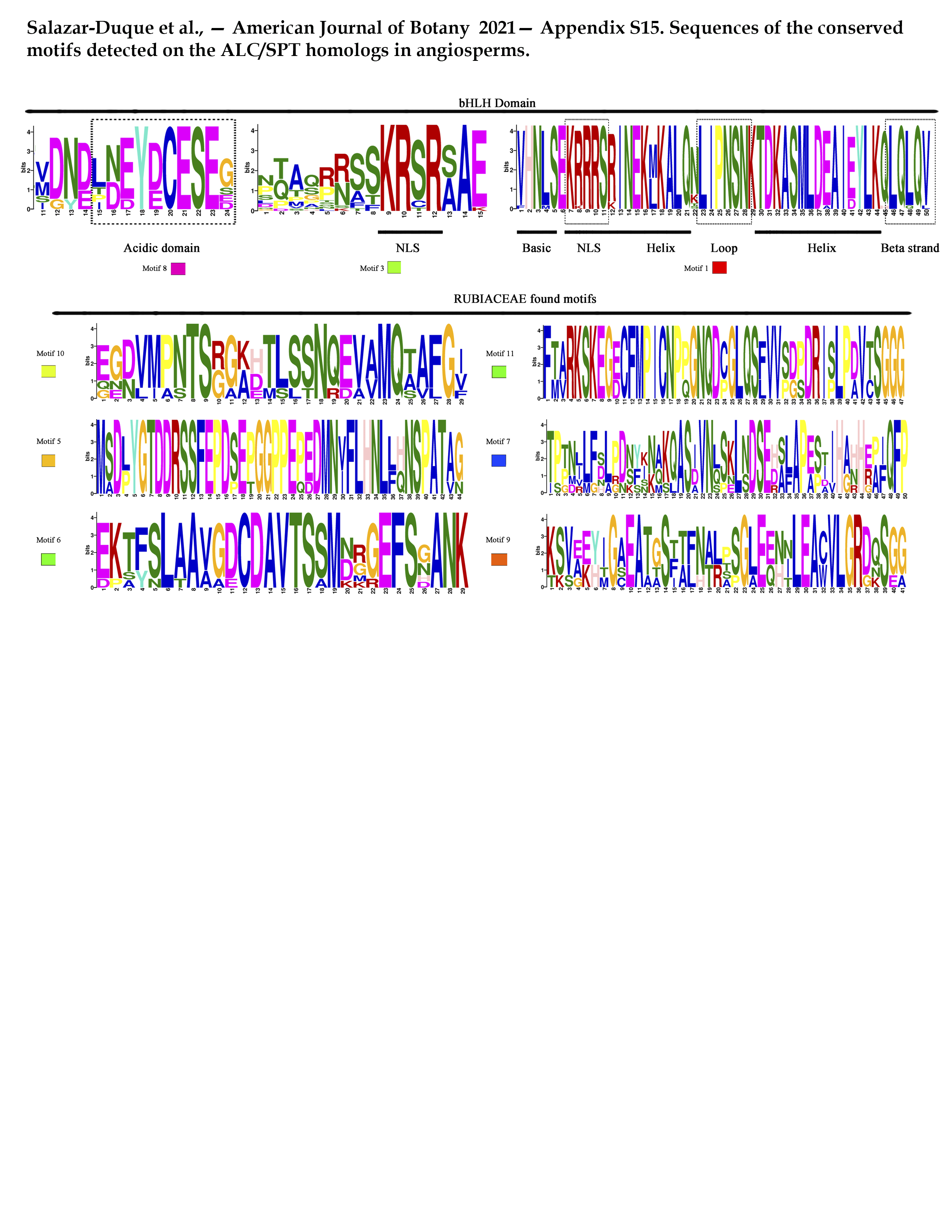

Supplement: Supplementary file 15 — Appendix S15. Sequences of the conserved motifs detected on the ALC/SPT homologs in angiosperms. The bHLH domain follows Groszmann et al. ( 2011). Rubiaceae specific motifs are highlighted. Black lines point to highly conserved sites in these consensus sequences. [file AJB2-108-1838-s009.tif]

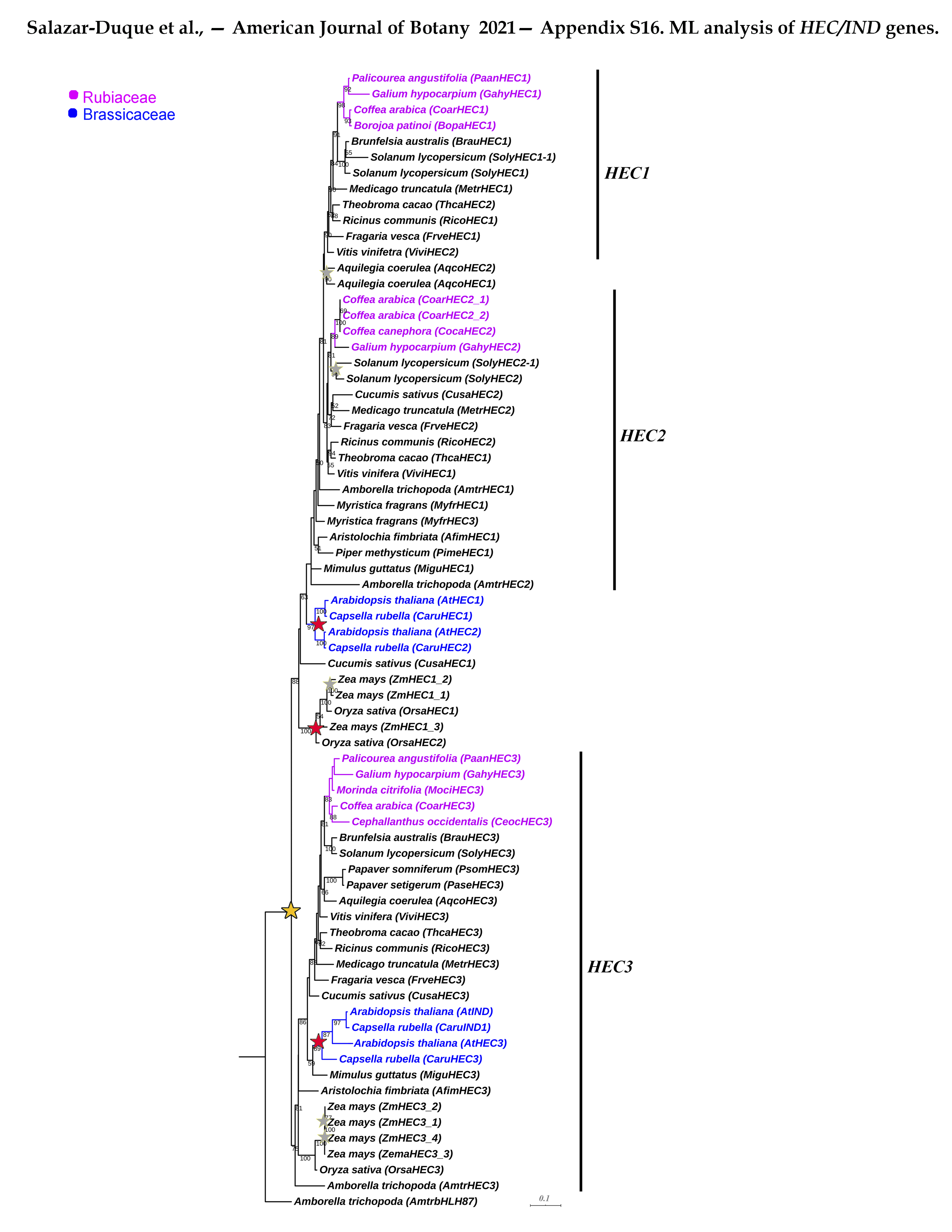

Supplement: Supplementary file 16 — Appendix S16. Maximum likelihood analysis of HEC/IND genes. Duplication events are indicated by the starts. Star and branch colors follow the same conventions indicated in Figure 2. Ultra‐Fast Bootstrap values are shown at nodes. [file AJB2-108-1838-s019.tif]

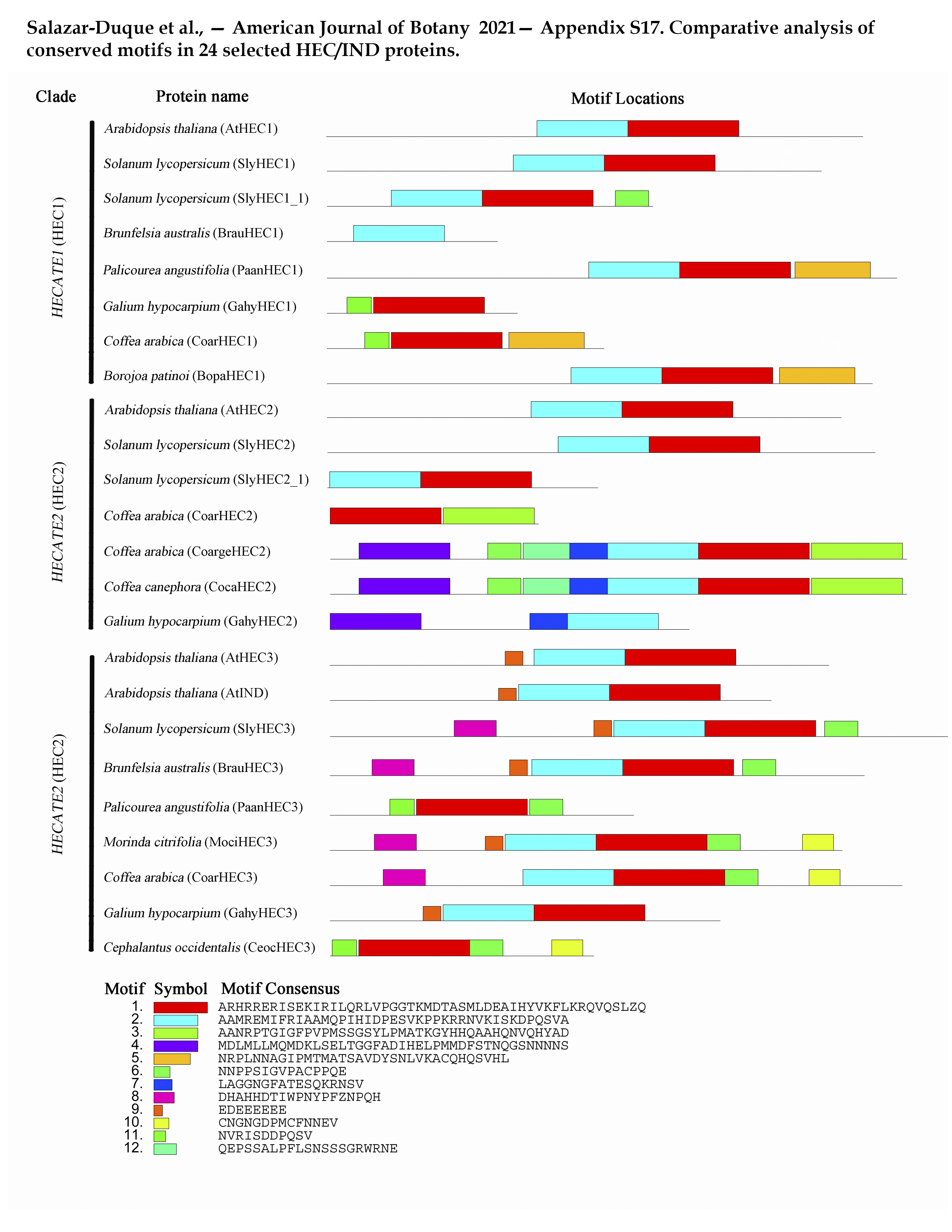

Supplement: Supplementary file 17 — Appendix S17. Comparative analysis of conserved motifs in 24 selected HEC/IND proteins. All the conserved motifs were identified using MEME suite. Colored boxes indicate motifs 1 to 12. Protein names and combined probability values are shown on the left. [file AJB2-108-1838-s016.tif]

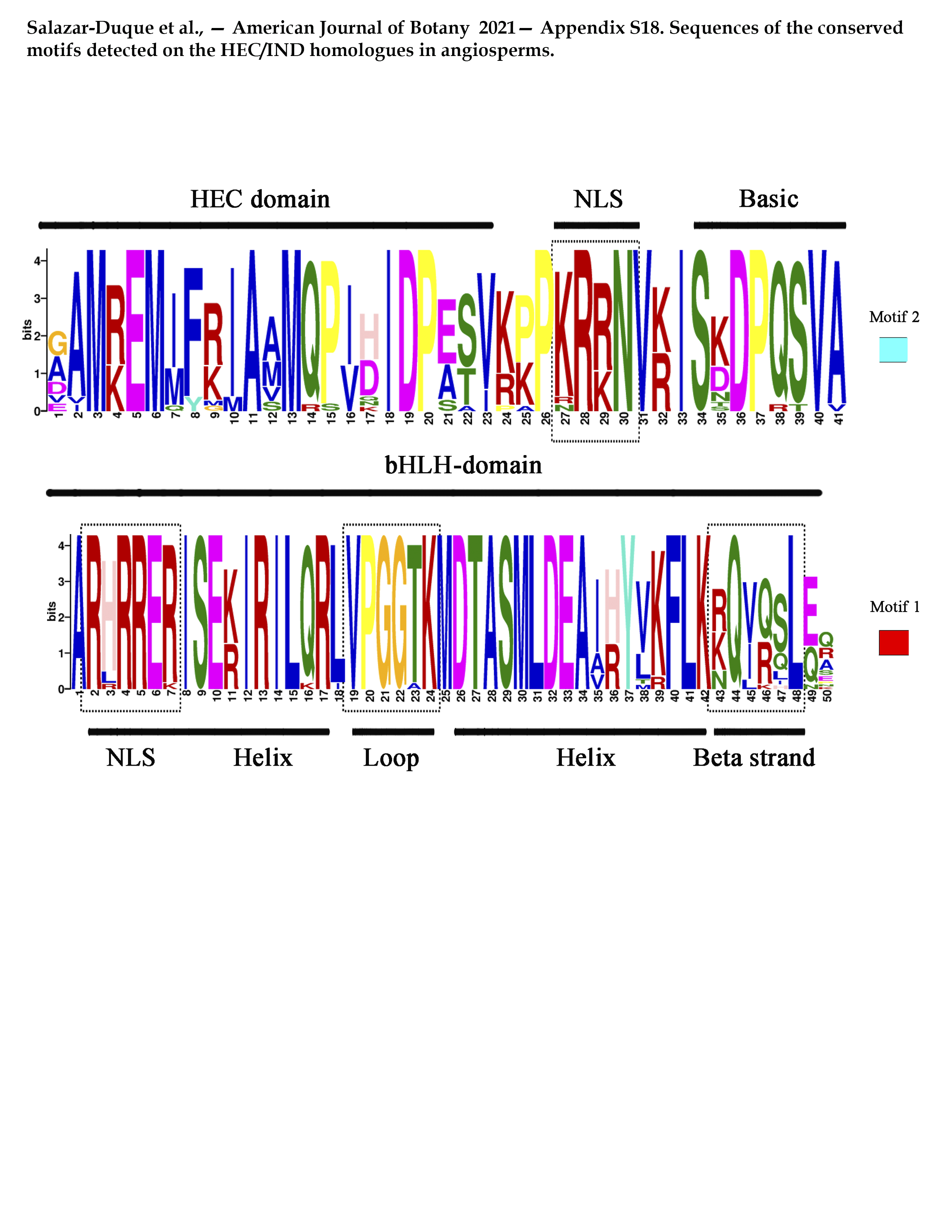

Supplement: Supplementary file 18 — Appendix S18. Sequences of the conserved motifs detected on the HEC/IND homologues in angiosperms. Black line on the left in motif 1 of the bHLH domain shows the HEC domain identified by Kay et al. ( 2013), the conserved regions of bHLH, and its beta strand tale. Dashed rectangles indicate de NLS region inside of the Helix 1 in the bHLH domain. [file AJB2-108-1838-s010.tif]

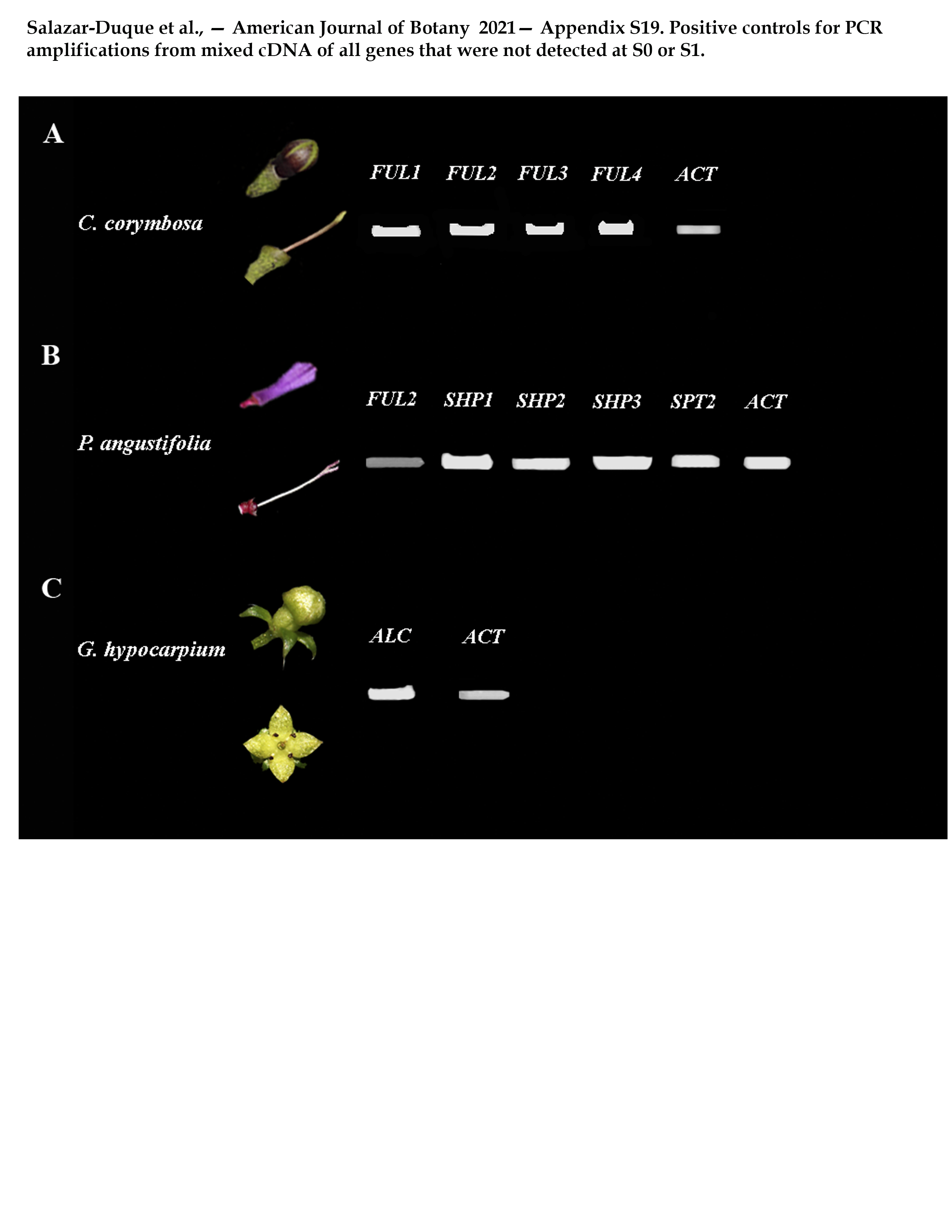

Supplement: Supplementary file 19 — Appendix S19. Positive controls for PCR amplifications from mixed cDNA of all genes that were not detected at S0 or S1. [file AJB2-108-1838-s003.tif]
